# LGBTQI Inclusive Cancer Care: A Discourse Analytic Study of Health Care Professional, Patient and Carer Perspectives

**DOI:** 10.3389/fonc.2022.832657

**Published:** 2022-05-10

**Authors:** Jane M. Ussher, Rosalie Power, Janette Perz, Alexandra J. Hawkey, Kimberley Allison

**Affiliations:** Translational Health Research Institute, School of Medicine, Western Sydney University, Sydney, NSW, Australia

**Keywords:** cancer care, cultural competence, LGBTQI, qualitative study, discourse analysis, healthcare professionals, patients and carers

## Abstract

**Background:**

Awareness of the specific needs of LGBTQI cancer patients has led to calls for inclusivity, cultural competence, cultural safety and cultural humility in cancer care. Examination of oncology healthcare professionals’ (HCP) perspectives is central to identifying barriers and facilitators to inclusive LGBTQI cancer care.

**Study Aim:**

This study examined oncology HCPs perspectives in relation to LGBTQI cancer care, and the implications of HCP perspectives and practices for LGBTQI patients and their caregivers.

**Method:**

357 oncology HCPs in nursing (40%), medical (24%), allied health (19%) and leadership (11%) positions took part in a survey; 48 HCPs completed an interview. 430 LGBTQI patients, representing a range of tumor types, sexual and gender identities, age and intersex status, and 132 carers completed a survey, and 104 LGBTQI patients and 31 carers undertook an interview. Data were analysed using thematic discourse analysis.

**Results:**

Three HCP subject positions – ways of thinking and behaving in relation to the self and LGBTQI patients – were identified:’Inclusive and reflective’ practitioners characterized LGBTQI patients as potentially vulnerable and offered inclusive care, drawing on an affirmative construction of LGBTQI health. This resulted in LGBTQI patients and their carers feeling safe and respected, willing to disclose sexual orientation and gender identity (SOGI) status, and satisfied with cancer care. ‘Egalitarian practitioners’ drew on discourses of ethical responsibility, positioning themselves as treating all patients the same, not seeing the relevance of SOGI information. This was associated with absence of LGBTQI-specific information, patient and carer anxiety about disclosure of SOGI, feelings of invisibility, and dissatisfaction with healthcare. ‘Anti-inclusive’ practitioners’ expressed open hostility and prejudice towards LGBTQI patients, reflecting a cultural discourse of homophobia and transphobia. This was associated with patient and carer distress, feelings of negative judgement, and exclusion of same-gender partners.

**Conclusion:**

Derogatory views and descriptions of LGBTQI patients, and cis-normative practices need to be challenged, to ensure that HCPs offer inclusive and affirmative care. Building HCP’s communicative competence to work with LGBTQI patients needs to become an essential part of basic training and ongoing professional development. Visible indicators of LGBTQI inclusivity are essential, alongside targeted resources and information for LGBTQI people.

## 1 Introduction

Attention to the nature and impact of interactions between oncology healthcare professionals (HCPs) and lesbian, gay, bisexual, transgender (trans), queer, and intersex (LGBTQI) patients is increasing (1, 2). This follows recognition of the vulnerability and unique concerns of this underserved patient population, who have a high rate of unmet needs (3–6). LGBTQI individuals are at higher risk of cancer compared with the general population (4, 5, 7), but are less likely to engage in cancer screening or have a regular healthcare provider (8–10). More specifically, LGBTQI patients report high levels of dissatisfaction with cancer healthcare (3, 11), barriers to accessing cancer services (3), and difficulties in communication with HCPs (4, 12). This includes heteronormative assumptions on the part of HCPs, or overt HCP hostility and discrimination, leading to LGBTQI patient anxiety associated with disclosure of sexual orientation or gender identity (SOGI) (4, 12–14). The absence of LGBTQI-specific cancer information or support serves to render LGBTQI people and their carers invisible (4, 15). Unique psychosocial challenges are often not acknowledged or addressed by HCPs, including sexual concerns related to same-gender relationships (15–17), the impact of minority stress (18), absence of support from biological family (6, 19), and the specific concerns of trans and intersex individuals (20–22). As a result, many LGBTQI individuals report anxiety, isolation and frustration throughout their cancer care (3, 4), leading to higher rates of distress (20, 23, 24) and lower quality of life (18), compared with the general cancer population.

Awareness of the unmet needs of LGBTQI patients has led to calls for HCPs to be trained in the practice of inclusive and affirmative cancer care (3, 5, 6), variously described as cultural competence (2, 25–27), cultural humility (28), or cultural safety (29). Whilst these concepts were originally developed to address health inequities experienced by indigenous people (30), they are increasingly being applied to other marginalised populations (29), including LGBTQI people (31). Culturally competent healthcare involves cultural awareness, cultural knowledge and cultural skill, applied in all areas of practice, including the clinical setting, administration, policy development, and HCP education (32). The concept of cultural humility places less emphasis on acquisition of specific communication skills, focusing on the ongoing commitment of HCPs to engage in self-reflection and to addressing power imbalances implicit in patient-HCP interactions through open and empathic supportive interactions (30, 33, 34). Cultural safety focuses on creating an environment within the healthcare system that is emotionally, socially and physically safe, with no actions taken to challenge or diminish the identities of an individual (30, 35). HCPs who practice cultural safety are responsive to the personal circumstances and cultural needs of their patients and are free from bias and discrimination in a way that the patients experience as safe (30, 35). Inclusive and affirmative LGBTQI cancer care involves these three complementary concepts: cultural competence, cultural humility and cultural safety.

Consideration of oncology HCPs’ perspectives is central to identifying barriers and facilitators of the provision of inclusive and affirmative LGBTQI cancer care (2). Greater knowledge of LGBTQI healthcare needs is associated with positive attitudes and intentions to offer inclusive and affirmative care for LGBTQI cancer patients (36–38). However, surveys of oncology physicians (1, 2, 37), radiation therapists (39), nurses and other advanced care professionals (36, 38, 40, 41) consistently report low levels of knowledge about LGBTQI patients. This includes lack of knowledge about cancer risk factors and psychosocial vulnerabilities specific to LGBTQI people (1, 38, 40), and feeling ill-informed about LGBTQI cancer patients’ unique needs (2, 39, 42). Lack of knowledge has implications for HCP confidence and comfort in treating LGBTQI patients (1, 2, 37), with sexual health (43), fertility (44), and the needs of trans (1, 14, 45) and intersex patients (41) being areas where communication challenges are most likely. Moreover, even the majority of HCPs who report being comfortable treating LGBTQI patients in cancer care surveys (1, 2, 40, 42), report a desire for education and training (1, 36, 37, 41) focused on the needs and best ways of working with the LGBTQI population.

A number of strategies and models have been developed to raise HCPs awareness of LGBTQI patients, with the aim of improving communicative competence (4, 6, 28, 42). Such models operate on the premise that if HCPs are knowledgeable of the unique needs of LGBQTI patients with cancer, and provided with guidelines on how to communicate appropriately, they will do so. Underpinning these models and strategies is a ‘one size fits all’ approach, which assumes a universality of context and complexity in HCP-LGBTQI patient interactions. This does not account for HCPs often engaging in negotiating information provision and communication on a case-by-case basis, in a context that is shaped by the interaction of structural, personal and socio-cultural constraints (43). Little attention has been paid to the ways in which socio-cultural constructions of LGBTQI people, and the subject positions adopted by HCPs in relation to LGBTQI patients, inhibit or facilitate the provision of affirmative and inclusive cancer care, and the impact of HCP subject positions on patients. This is the focus of the present study.

It has been recognized that it is important to understand the “nuances of communication” that occur between HCPs and LGBTQI patients, in particular challenges in when and how to address sexual orientation and gender identity (SOGI) disclosure (14), in order to develop effective and targeted communicative competence interventions for HCPs. It is also important to be cognizant of the intersection of identities of LGBTQI patients, including age, sexual orientation, gender identity and cultural background, which may influence healthcare interactions (20). With the exception of two mixed-methods studies that included open-ended survey responses (14, 45), previous research on HCP perspectives on LGBTQI cancer care has utilized quantitative survey methods. There is a need for in-depth qualitative methods, including interviews and open-ended survey responses, to develop deeper, richly textured insight into the subject positions adopted by HCPs in relation to LGBTQI cancer care, and the implications of HCP positioning and practice for patients (43, 45). There is also a need for research that includes the perspectives of medical, nursing and allied HCPs, as well as those in leadership positions, in a range of clinical settings (37), reflecting the multidisciplinary model of care cancer (46). Most published studies to date focus on USA-based oncology physicians (1, 2, 37, 45), with a minority including oncology social workers (42), advanced healthcare practitioners (36), or nurses (38).

Research on the perspectives of LGBTQI cancer patients has identified that in the absence of visible indicators (e.g., rainbow flag) that health care settings or individual HCPs are inclusive and affirmative, many LGBTQI people fear that they will face HCP hostility and discrimination, and be offered substandard cancer care (4, 12, 13, 47, 48). Patients who experience negative HCP reactions can experience distress and disengagement with cancer care (4, 12, 13). Previous research on patient perspectives on interactions with oncology HCPs has focused on cisgender adults with breast or prostate cancer, who identify as lesbian, gay or bisexual (4, 6, 20). There is need for research that includes the perspectives of LGBTQI individuals across a wider range of cancer types, adolescents and young adults (AYAs), as well as transgender, gender diverse and intersex people with cancer (20). There is also a need to include the perspectives of partners and other caregivers, an understudied group in cancer research who report high rates of distress (49, 50). For LGBTQI people, caregiving is often provided by ‘chosen family’ (51), which includes intimate partners and friends (19).

The aim of the present study was to examine the construction and experience of LGBTQI cancer care from the perspective of HCPs, LGBTQI patients and their caregivers, using qualitative methods. The research questions were: What subject positions do oncology HCPs occupy in relation to the provision of care to LGBTQI people? What are the implications of HCP positions for LGBTQI patients and their caregivers?

### 1.1 Summary of Key Acronyms

AYA, Adolescents and young adults

HCP, Healthcare professionals

iKT, Integrated knowledge translation

LGB, Lesbian, gay and bisexual

LGBQ, Lesbian, gay, bisexual and queer

LGBT, Lesbian, gay, bisexual and transgender

LGBTQI, Lesbian, gay, bisexual, transgender, queer and/or intersex

SGM, Sexual and gender minority

SOGI, Sexual orientation and gender identity

TGD, Transgender and gender diverse

## 2 Methods

### 2.1 Study Design

**Figure 1:**
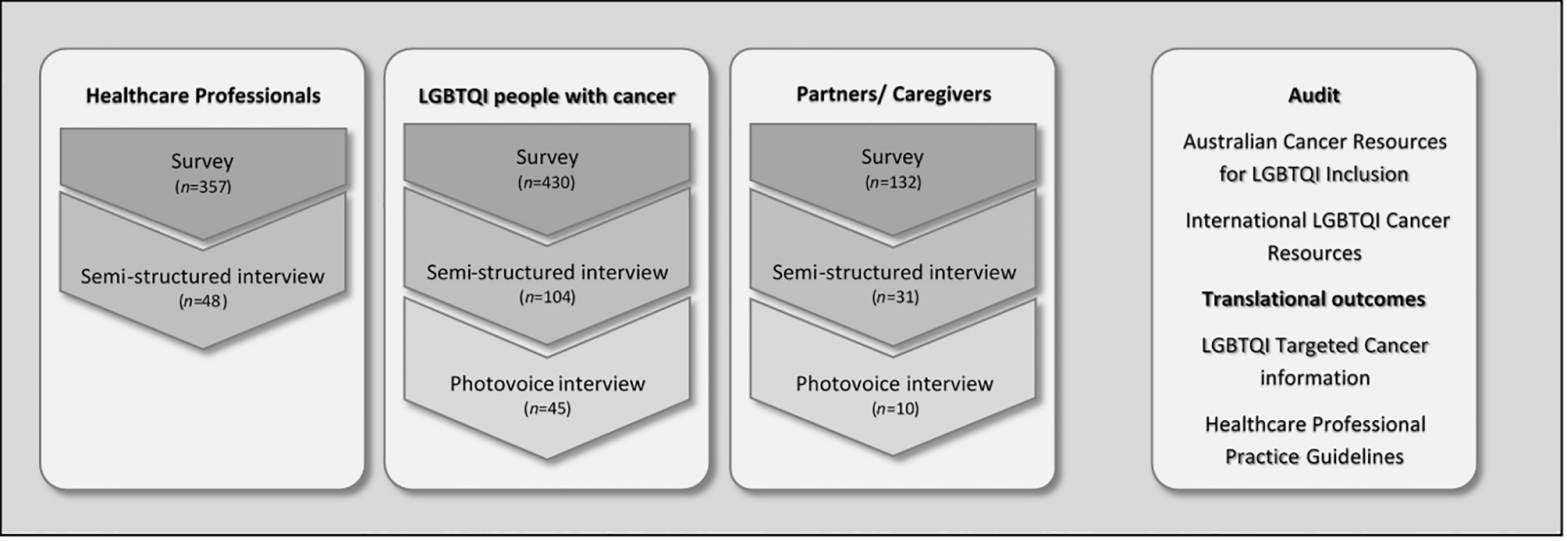
The Out with Cancer Study overall design.

This cross-sectional study was part of a broader mixed-methods project, the ‘Out with Cancer Study’ (41, 52). The overall project examined LGBTQI cancer and cancer care from the perspectives of LGBTQI patients and their caregivers, and HCPs; audited Australian cancer resources for LGBTQI cultural competence and reviewed international LGBTQI cancer resources; and produced targeted LGBTQI cancer resources and healthcare professional practice guidelines (**Figure 1**). This paper presents the analysis of qualitative survey responses and interviews related to interactions between oncology HCPs with LGBTQI patients and their partners and other caregivers (carers). The survey facilitated data collection from a large group of LGBTQI individuals, including a range of sexual and gender identities, ages, and tumor types, with the interviews allowing for in-depth exploration of experiences in a selected sub-section of survey respondents (**Figure 1**).

In the study design, data collection, analysis and dissemination, we drew on principles of integrated knowledge translation (iKT), a dynamic process of collaboration between researchers and knowledge users to achieve actionable research outcomes (53). Following principles of iKT, a steering committee comprising LGBTQI people with cancer, cancer HCPs and representatives from LGBTQI health and cancer support organizations were actively involved throughout all stages of the study. Ethics approval was provided by Western Sydney University Human Research Ethics Committee (H12664). All participants provided informed consent.

### 2.2 Participants and Recruitment

#### 2.2.1 Health Care Professionals

HCPs providing services to people with cancer and their carers were eligible to participate in this study. Participants were recruited through targeted advertisements on social media (e.g., Facebook, Twitter), *via* professional networks (e.g., Clinical Oncology Society of Australia, Cancer Nursing Society of Australia) and through cancer-related community organizations. We specifically targeted oncology medical practitioners, nurses, allied health professionals (e.g., social workers, psychologists, occupational and physiotherapists) and individuals working in leadership roles in cancer care, health and preventative agencies such as support group leaders, program/service managers and consumer representatives/advocates. The study procedures and quantitative survey results from HCPs have been published elsewhere (41). Briefly, a sample of 357 HCPs working with people with cancer in nursing (40%), medical (24%), allied health (19%) and leadership (11%) positions, took part in an online anonymous survey. The majority (88%) were based in Australia, with a mean age of 47 (SD =10), and an average of 14 years’ experience in cancer care (see **Table 1** for HCP demographics). Survey participants were invited to volunteer for a follow-up interview to examine their perspectives on LGBTQI cancer care in more detail. Of those who agreed to participate, a subset of 48 HCPs was selected, representing a range of professional backgrounds and gender identities. The one-to-one interviews lasted between 30 to 60 minutes, and were recorded. The study was open to HCPs from May 2020 to March 2021.

**Table 1:** Sociodemographic and Professional Characteristics of Participating Health Care Professionals.

Demographic/Professional characteristic	Survey participants	Interview participants
	*M (SD)*, range	*M (SD)*, range
Age (years) (n = 356)	47.29 (12.45),	45.94 (13.04),
	22-82	24-68
Time working in cancer care (years) (n = 303)	14.31 (10.21), 0.33-45	13.15 (9.89), 0.50-40
	*n* (%)	*n* (%)
Gender (n = 357)		
Female	278 (77.9%)	36 (75.0%)
Male	76 (21.3%)	12 (25.0%)
Non-binary	3 (0.8%)	0
Ethnicity (n = 352)		
Caucasian	305 (85.4%)	42 (87.5%)
Asian	22 (6.2%)	2 (4.2%)
Middle Eastern/African	6 (1.7%)	3 (6.3%)
Mixed background	8 (2.2%)	1 (2.1%)
Other/unclear background^1^	11 (3.1%)	0
LGBTQI+ themselves (n = 328)		
Yes	60 (18.3%)	18 (37.5%)
No	264 (80.5%)	30 (62.5%)
Prefer not to answer	4 (1.2%)	0
Has LGBTQI+ family (n = 328)		
Yes	135 (41.2%)	25 (52.1%)
No	191 (53.5%)	22 (45.8%)
Prefer not to answer	2 (0.6%)	1 (2.1%)
Has LGBTQI+ friend/s (n = 328)		
Yes	300 (91.5%)	47 (97.9%)
No	28 (7.8%)	1 (2.1%)
Country (n = 357)		
Australia	315 (88.2%)	44 (91.7%)
United States of America	17 (4.8%)	0
United Kingdom	10 (2.8%)	3 (6.3%)
New Zealand	5 (1.4%)	0
Canada	3 (0.8%)	1 (2.1%)
Other	7 (2.0%)	0
Professional discipline (n = 356)		
Medical	87 (24.4%)	12 (25.0%)
Nursing	142 (39.9%)	15 (31.3%)
Allied health	69 (19.4%)	15 (31.3%)
Leadership	38 (10.7%)	4 (8.3%)
Other^2^	20 (5.6%)	2 (4.2%)
Workplace location (n = 355)		
Urban	247 (69.2%)	38 (79.2%)
Regional	85 (26.6%)	9 (18.8%)
Rural	9 (2.5%)	1 (2.1%)
Remote	4 (1.1%)	0
Healthcare sector*		
Public	230 (64.4%)	29 (60.4%)
Private	72 (20.2%)	10 (20.8%)
Primary healthcare	9 (2.5%)	0
Community-based	11 (3.1%)	2 (4.2%)
Not for profit	88 (24.6%)	16 (33.3%)
Something else	24 (6.7%)	2 (4.2%)
Number of patients seen per week (n = 318)		
0-25	189 (59.4%)	23 (48.9%)
26-50	75 (23.6%)	12 (25.5%)
51-75	29 (8.1%)	9 (19.1%)
76+	25 (7.9%)	3 (6.4%)
Age groups seen* (n = 320)		
Paediatric	17 (5.3%)	1 (2.1%)
Adolescent and young adult	86 (26.9%)	18 (38.3%)
Adult	279 (87.2%)	39 (83.0%)
Older adult/elderly	177 (55.3%)	29 (61.7%)
Estimated proportion of patients who are LGBTQI+ (n = 317)		
None	29 (9.1%)	0
<5%	154 (48.6%)	24 (51.1%)
6-10%	58 (18.3%)	13 (27.7%)
11-15%	10 (3.2%)	1 (2.1%)
16-20%	4 (1.3%)	0
> 20%	2 (0.6%)	2 (4.3%)
Unsure	57 (18.0%)	7 (14.9%)
N/A	3 (0.9%)	0
Had formal education about healthcare needs of…* (n = 355)		
Sexuality diverse people	96 (27.0%)	23 (47.9%)
Trans and gender diverse people	74 (20.8%)	18 (37.5%)
People born with an intersex variation	52 (14.6%)	11 (22.9%)

*Participants could select multiple options for questions about healthcare sector, age groups seen, and LGBTQI healthcare training.

^1^Ethnicity Other/unclear background: Latin American (n = 4), Jewish (n = 3), Aboriginal (n = 1), not clearly described (n = 3).

^2^Professional background - Other: Research (n = 7), administration (n = 3), dentistry (n = 1), paralegal (n = 1), education/training (n = 1), none/retired (n = 7).

#### 2.2.2 LGBTQI People With Cancer and Their Partners/Carers

A sample of 430 LGBTQI people who currently or previously had cancer (patients) with a range of tumor types and 132 partners or other caregivers (carers), aged 15 years and older, took part in an online anonymous survey, the details and results of which are published elsewhere (54). **Table 2** contains demographic details of the survey participants, by patients and carers. The majority of patients were living in Australia (72.3%), Caucasian (85.2%), and identified themselves as lesbian, gay or homosexual (73.7%), with 10.9% identifying as bisexual, and 10.5% as queer. Greater diversity was evident in participants’ gender identities: 50.2% were cis women, 33.7% cis men, 16.1% TGD. Thirty-one (7.2%) participants reported intersex variation. The average patient age was 52.5 years (SD 15.7), with 22% in the AYA age-group (age 16-39).

**Table 2:** Demographic and cancer characteristics of LGBTQI patients and carers - Survey Participants.

Demographic/Cancer Characteristic	Patient Survey		Carer Survey			
	Patients		Carers		Patients carer for by carers^1^	
	*N*	*M (SD)*, range	*N*	*M (SD)*, range	*N*	*M (SD)*, range
Age at time of study (years)	429	52.5 (15.7), 16-92	132	50.2 (17.0), 15-76	–	–
Age at diagnosis (years)	363	46.3 (15.3), 1-79	126	42.8 (16.6), 0-70	120	50.3 (15.6), 1-92
	*N*	*n* (%)	*N*	*n* (%)	*N*	*n* (%)
Country	430		132		–	–
Australia		311 (72.3%)		93 (70.5%)		
United States of America		62 (14.4%)		14 (10.6%)		
United Kingdom		29 (6.7%)		9 (6.8%)		
New Zealand		8 (1.9%)		6 (4.5%)		
Canada		7 (1.6%)		4 (3.0%)		
Other		13 (3.0%)^2^		6 (3.6%)^3^		
Gender	430		132		132	
Cis female		216 (50.2%)		83 (62.9%)		90 (68.2%)
Cis male		145 (33.7%)		26 (19.7%)		36 (27.3%)
Non-binary		34 (7.9%)		16 (12.1%)		2 (1.5%)
Trans female		13 (3.0%)		5 (3.8%)		1 (0.8%)
Trans male		8 (1.9%)		2 (1.5%)		0
Different or multiple identities		14 (3.3%)^4^		0		3 (2.3%)
Sexuality	430		132		131	
Lesbian, gay or homosexual		317 (73.7%)		95 (72.0%)		81 (61.8%)
Bisexual or pansexual		47 (10.9%)		17 (12.9%)		5 (3.8%)
Queer		45 (10.5%)		12 (9.1%)		5 (3.8%)
Straight or heterosexual		10 (2.3%)		5 (3.8%)		33 (25.2%)
Different or multiple identities		11 (2.6%)		3 (2.3%)		1 (0.8%)
Not sure		–		–		6 (4.6%)
Intersex variation	430		132		132	
Yes		31 (7.2%)		5 (3.8%)		0
No		388 (90.2%)		127 (96.2%)		127 (96.2%)
Prefer not to answer		11 (2.6%)		0		0
Not sure		–		–		5 (3.8%)
Race/ethnicity	425		132		–	–
Caucasian		362 (85.2%)		109 (82.6%)		
Asian		11 (2.6%)		5 (3.8%)		
Australian Aboriginal, Torres Strait Islander or Maori		9 (2.1%)		4 (3.0%)		
Mixed background		19 (4.5%)		6 (4.5%)		
Other/unclear background		24 (5.6%)^5^		8 (6.1%)^6^		
Education	422		131		–	–
Less than secondary		10 (2.4%)		7 (5.3%)		
Secondary		45 (10.7%)		17 (13.0%)		
Some post-secondary		55 (13.0%)		9 (6.9%)		
Post-secondary		312 (73.9%)		98 (74.8%)		
Location	429		132		–	–
Urban		234 (54.5%)		69 (52.3%)		
Regional		145 (33.8%)		48 (36.4%)		
Rural or remote		50 (11.7%)		15 (11.4%)		
Relationship to PWC	–	–	132		–	–
Partner/ex-partner				84 (63.6%)		
Family				31 (23.5%)		
Friend				12 (9.1%)		
Different relationship				3 (2.3%)		
Multiple PWCs/relationships				2 (1.5%)		
Cancer diagnosis (first)	370		–	–	129	
Brain		11 (3.0%)				9 (7.0%)
Breast		90 (24.3%)				37 (28.7%)
Cervical		11 (3.0%)				4 (3.1%)
Colorectal		17 (4.6%)				8 (6.2%)
Head/neck		14 (3.8%)				10 (7.8%)
Leukaemia		17 (4.6%)				5 (3.9%)
Lymphoma		24 (6.5%)				6 (4.7%)
Ovarian		17 (4.6%)				13 (10.1%)
Prostate		59 (15.9%)				8 (6.2%)
Skin		25 (6.8%)				3 (2.3%)
Uterine		23 (6.2%)				4 (3.1%)
Other		58 (15.7%)^7^				19 (14.7%)^8^
Not sure or unknown		4 (1.1%)				3 (2.3%)
Cancer stage	369		–	–	129	
Localised		228 (61.8%)				55 (42.6%)
Regional		88 (23.8%)				43 (33.3%)
Distant/metastatic		32 (8.7%)				23 (17.8%)
N/A (e.g. blood cancer)		5 (1.4%)				1 (0.8%)
Not sure or unclear		16 (4.3%)				7 (5.4%)
Subsequent cancers*	370		–	–	129	
Recurrence		57 (15.4%)				30 (23.3%)
New primary cancer		40 (10.8%)				20 (15.5%)
Treatment status	370		–	–	129	
No treatment yet		37 (10.0%)				5 (3.9%)
On active curative treatment		37 (10.0%)				14 (10.9%)
On maintenance treatment		60 (16.2%)				19 (14.7%)
In remission/completed treatment		217 (58.6%)				35 (27.1%)
Receiving palliative care (no further active reatment)		4 (1.1%)				2 (1.6%)
Deceased		–				51 (39.5%)
Not sure, unclear, or multiple		8 (2.2%)				3 (2.3%)

^1^ Key demographic and cancer characteristics of the patients who carers cared for.

^2^ Austria (n=4), Bahrain, Chad, Costa Rica, Denmark, Germany, Morocco, Poland, Russian Federation, Serbia (n=1 each).

^3^ Belize (n=2), Argentina, Lebanon, Germany, Uganda (n=1 each).

^4^ Intersex (n=4), female with fleeting genderfluid moments, intersex woman, intersex nonbinary woman, female but questioning, trans (n=1 each).

^5^ Jewish (n = 9), Hispanic/Latine (n = 4), Middle Eastern, Native American, Romani (n=1 each), not clearly described (n = 8).

^6^ Hispanic/Latine, Jewish (n = 2 each), African, Native American (n=1 each), not clearly described (n = 2).

^7^ Sarcoma (n=9), kidney, testicular (n=8 each), bladder, thyroid (n=6 each), lung (n=5) anal, pancreatic (n=4 each), liver (n=2), something else (n=6).

^8^Lung (n=7), bladder, liver, pancreatic (n=2 each), kidney, mesothelioma, pseudo myxoma perotini, sarcoma, stomach, thymus (n=1 each).

*Participants could selected multiple options, if applicable.

Survey participants were invited to take part in an interview for the purpose of understanding their experiences in greater depth. A subset of 104 LGBTQI patients and 31 partners/carers, representing a cross-section of participants in gender, sexuality, age and tumor type, completed a 60-minute interview. **Table 3** provides a demographic breakdown of interview participants, by gender, sexuality and intersex status. The study was open internationally, although recruitment focused on Australia and other English-speaking countries such as the USA, UK, New Zealand and Canada. Participants were recruited through social media (e.g., Facebook, Twitter, Instagram), cancer and LGBTQI community organizations (including the study partner organizations), cancer research databases (e.g., Register 4, ANZUP), LGBTQI community events (e.g., Sydney Gay and Lesbian Mardi Gras) and cancer support groups. The study was open to LGBTQI patients and their partners/carers from September 2019 to September 2021.

**Table 3:** LGBTQI patient and carer interview participants by sexuality, gender and intersex status.

	Patient n,%	Carer n,%
Gender		
Cis female	48 (46.2%)	18 (58.1%)
Cis male	42 (40.4%)	6 (19.4%)
TGD	11 (10.6%)	6 (19.4%)
Different identity	3 (2.9%)	1 (3.2%)
Sexuality		
Lesbian, gay or homosexual	86 (82.7%)	19 (61.3%)
Bisexual	5 (4.8%)	3 (9.7%)
Queer	9 (8.7%)	6 (19.4%)
Straight or heterosexual	1 (1.0%)	0 (0.0%)
Different or multiple identities	3 (2.9%)	3 (9.7%)
Intersex variation		
Yes	3 (2.9%)	1 (3.2%)
No	100 (96.2%)	29 (93.5%)
Prefer not to answer	1 (1.0%)	1 (3.2%)

TGD, trans and gender diverse.

### 2.3 Measures

#### 2.3.1 Qualitative Survey Items

The HCP survey (41) assessed attitudes toward LGBTQI cancer care, knowledge of LGBTQI health needs and LGBTQI inclusive practice behaviors. At the end of each section, HCP participants were asked to provide written responses to the open-ended question, “is there anything you would like to tell us about your answers to these questions?”. The LGBTQI patient survey assessed demographics, minority stress, disclosure, satisfaction with care, health literacy, end of life care issues, social support and relationships and sexual, physical and emotional wellbeing [described in (54)]. The carer survey assessed the same items, with the addition of items about caregiving experiences. At the end of most quantitative items, LGBTQI patients and partners/carers were asked to provide written responses to the open-ended question, “is there anything you would like to tell us about this issue?”, with responses ranging from one sentence to 15 sentences, with an average of 2 sentences. This paper focuses on qualitative responses to items on HCP interactions and the provision of cancer care, across participant groups.

#### 2.3.2 Semi-Structured Interviews

Semi-structured interviews with HCPs, LGBTQI patients and carers were completed over the telephone or online using videoconferencing software, depending on the preference of the participant. All interviews were audio-recorded and transcribed verbatum. Healthcare professionals were asked about their experiences providing care for LGBTQI patients, including how they identified LGBTQI patients, how well their workplaces were meeting the needs of LGBTQI patients and carers, and what they considered were important issues for LGBTQI patients and carers. LGBTQI patients and carers were asked about their experiences of cancer care, including interactions with HCPs, decision-making pertaining to disclosure of their LGBTQI status and the consequences of this for their cancer care; the impact of cancer on their lives, including on their identities; relationships and sexual wellbeing; support networks and experiences of finding information as an LGBTQI cancer patient. This paper focuses on HCP interactions and disclosure of LGBTQI status in cancer care.

### 2.4 Data Analysis and Theoretical Framework

Thematic discourse analysis or decomposition (55–57) was used to examine the qualitative survey responses and interviews. This analytic technique combines post-structuralist discursive approaches (58, 59) with thematic analysis (60), informed by the notion that meanings are socially formed through discourse (61). In this context, discourse refers to a ‘set of statements that cohere around common meanings and values… (that) are a product of social factors, powers and practices, rather than an individual’s set of ideas’ [(62), p.231]. Discourse analysis focuses on the subject positions that are taken up in talk, and their consequences in interactions for the self and others, including the way someone speaks or is spoken to, how a speaker describes herself and others, and the broader social discourse that a speaker draws upon (63, 64). Once a person takes up a particular subject position, they see the world from the vantage point of that position, influenced by the “particular images, metaphors, storylines and concepts which are made relevant within the particular discursive practice in which they are positioned” (61). Subject positions are not fixed, and are not properties of the individual, which means that participants may adopt more than one subject position, or move between subject positions (61). The possibility of choice is implicitly present, because there are many potentially contradictory discursive practices in which each person could engage (61).

The focus of analysis in this paper is on the subject positions made available to oncology HCPs through discourse and the implications of these subject positions for LGBTQI patients and carers. The analysis was conducted using an inductive approach, with the development of discursive themes and identification of subject positions being data driven, rather than based on pre-existing research on HCP interactions with LGBTQI cancer patients and carers. HCP, patient and carer interviews were transcribed, verified for accuracy by reading the transcripts while listening to the audio-recording and then de-identified by replacing participant names with pseudonyms. Through a collaborative process with stakeholder committee members, a subset of interviews for HCPs, patients and carers were independently read and re-read to identify first-order codes within the HCP and patient/carer data sets that represented commonality across accounts, such as ‘lack of knowledge’, ‘discrimination’ (HCPs) ‘feeling unsafe’, ‘difficulties in communication’ (patients/carers). Each team member brought suggestions of the first order codes to the meeting and the final coding frames for HCPs and patients/carers were devised through a process of consensus. This included codes such as ‘culturally safe care, services and support’, ‘barriers to providing good LGBTQI care’, ‘experiences with LGBTQ patients and carers’ (HCPs); and ‘disclosure of identity’, ‘positive/negative interactions with HCPs’ (patients/carers). Open-ended survey and interview data were coded by four members of the research team using NVivo. Consistency in coding across codes and coders was checked by a senior member of the team. Coded data were read through and summarized in a tabular format to facilitate identification of commonalities in the data. The codes were then re-organized and grouped into discursive themes focused on subject positions adopted by HCPs in relation to LGBTQI cancer care and the implications of these subject positions for patients and carers. Themes were then refined through discussion, reorganized and, when consensus was reached, final themes and sub-themes developed. Throughout, the analysis was informed by an intersectional theoretical framework. This acknowledges the interaction and mutually constitutive nature of gender, sexual identity, age and other categories of difference in individual lives and social practices, and the association of these arrangements with health and wellbeing (65).

## 3 Results

**Figure 2:**
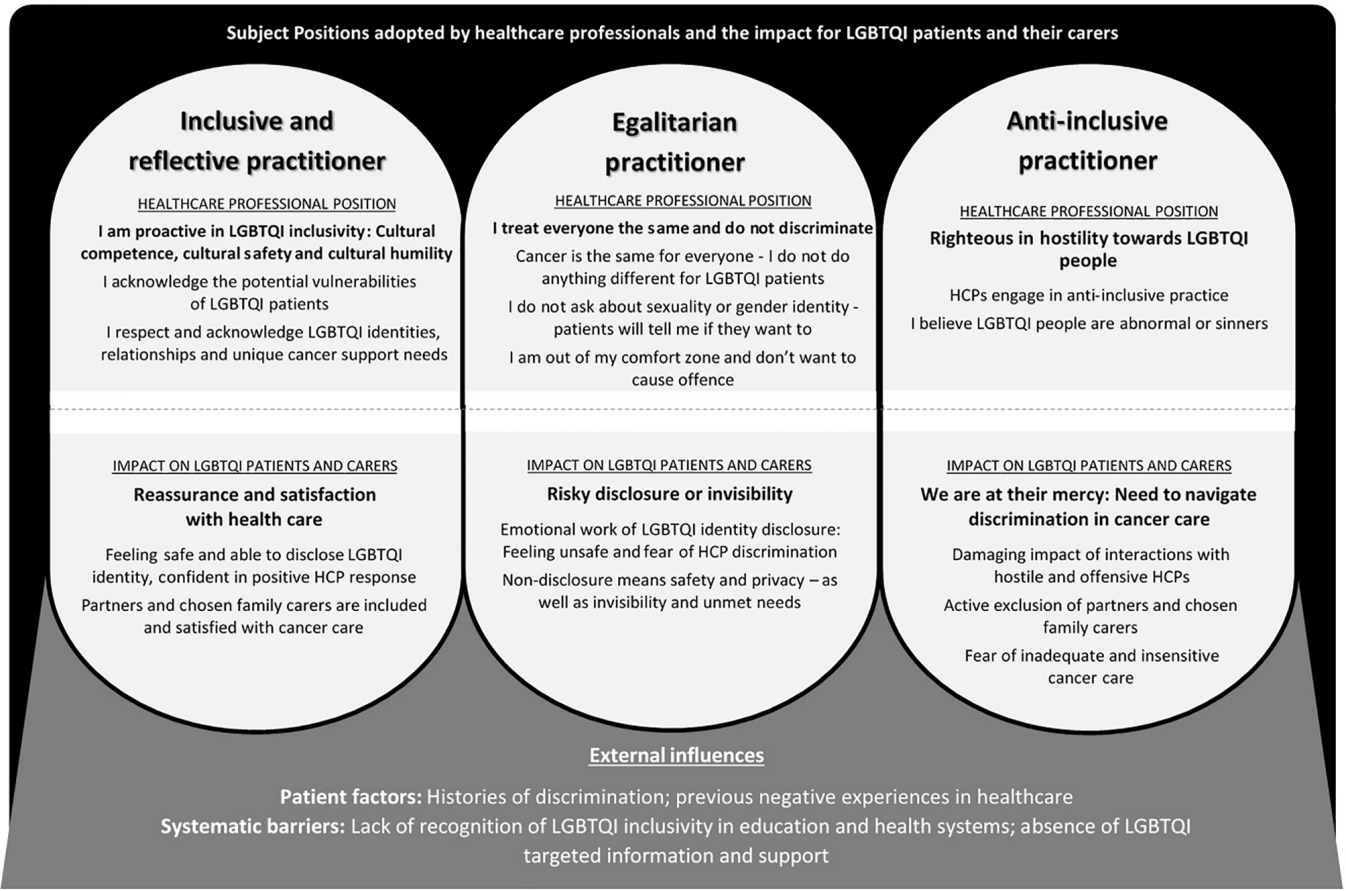
Subject Positions Adopted by Health Care Professionals and the Impact for LGBTQI Patients and their Carers.

Three subject positions adopted by HCPs were identified (**Figure 2**): Inclusive and Reflective practitioner; Egalitarian practitioner; and Anti-Inclusive practitioner. HCPs adopted these subject positions across the range of professional backgrounds, gender, sexual orientation and age groups. A number of HCPs adopted more than one subject position, with adoption of the Inclusive and Reflective Practitioner in some contexts and the Egalitarian Practitioner in others, identified. Each subject position had direct consequences for the positioning and experiences of LGBTQI patients and their carers (**Figure 2**).

In the presentation of results below, we outline each HCP subject position, followed by the patient and carer accounts of interactions with HCPs who adopted this subject position. Key demographic details are provided for longer quotes; Med= medical practitioner, Allied = allied health worker. LGBTQI patients and carers are identified by pseudonyms (interview participants) or “survey”, with demographic details of age, SOGI and intersex status, and cancer type provided for longer quotes (medical intervention = intervention to prevent cancer). In the patient/carer sections, participants who are carers are identified as such; all other quotes are from patients. For readability, demographic details for HCP and LGBTQI patient/carer short quotes are provided in **Table 4**, alongside a longer version of the quote for readability.

**Table 4:** Additional Quotes from Health Care Professionals, and LGBTQI Patients and Carers.

Inclusive and Reflective Practitioner
**I am Proactive in LGBTQI Inclusivity: HCPs Practice Cultural Competence, Safety and Humility.**
As a gender-diverse and queer person, when I go in, I don’t make those assumptions about my patients and when they talk about like, I’ll refer to their spouse or their partner, I don’t put those assumptions on them. And I think that makes queer patients a lot more comfortable because they don’t feel like they have to have the awkward correctional coming out. [Lane, Clinical Trials coordinator, 26, Queer, Non-binary] (1)#
They need to feel welcomed, they need to be able to feel that they can come out in health care without having to struggle over all these barriers. [Emily, Allied, 54, Lesbian] (2)
Establishing early contact and building report early and creating a safe space and thinking about hetero normative language is really important for AYAs. [Natasha, Allied, 30, Lesbian] (3)
To be seen by the health practitioner is really important. An aspect of respect, I think, is to respect people’s terminology and self-identification. Another aspect is really respecting and making the relationship or relationships visible. [Suzanne, Med, 40, Queer] (4)
If somebody is trans and they’ve grown out their hair, are they going to feel like they’ve taken a step backwards or something if they lose their hair? [Amelia, Nurse, 35, Lesbian] (5)
They would be having a different experience of it, because when we talk patients we talk about the impact it’s had on their body and also their sense of identity. And things like, weight loss or weight gain, it can very much impact on body image and identity for gay men. [Lexie, Allied, 27, Straight] (6)
This diversity of information should be considered mainstream and the norm rather than an exception to routine practice. For example, being able to give advice to a gay/bisexual man about factors influencing PSA testing, safe timing and approaches to resumption of anal sex after prostate radiotherapy [Survey, Allied, 62, Straight] (7)
The majority of our staff talk about using condoms for intercourse and don’t divulge into, you know, what about other types of barrier protection that’s not heterosexual penetrative sex, that doesn’t just focus on using a condom. [Jessica, Nurse, 38, Straight] (8)
I ask questions explicitly- that will tend to be how it will come up, if somebody has a same-sex partner, then it comes out that way in talking about what their support networks are. [ … ] So for me, it will come up whenever I get into sort of discussion about who somebody has in their life, who’s going to support them through their cancer diagnosis. [Brett, Med, 37, Gay] (9)
It’s essential that everybody assiduously takes note of the preferred gender pronouns and doesn’t dead name them. To be aware that sometimes the name in the medical notes is not the preferred name. And if you don’t know, ask, you know. [Suzanne, Med, 40, Queer] (10)
It’s quite daunting, for every patient but particularly for LGBTQI patients. Those kinds of things, like a poster or sticker or whatever it is, I think they make like a big difference for the communities. [Belinda, Med, 44, Lesbian] (11)
**Reassurance and Satisfaction with Health Care: LGBTQI Patient and Carer Perspectives on Inclusive and Reflective Practice**
Of my cancer-care doctors, my sexuality has mainly been treated as a non-issue. My GP is a gay man so it is openly discussed. My surgeon was welcoming to anyone I brought with me to appointments including my female partner. [Survey, 39, Queer, Breast] (12)
The nurses always refer to me as my kids mom and they even went out of their way to say to my kids, what’s this mum called and what do you call that mom. They interacted positively with my children, with my partner and with me. [Virginia, 48, Lesbian, Lymphoma] (13)
Because we live in a rural, small town area, where everyone knows everyone - I think we experience little discrimination, it helps with being respected. [Survey, Partner, 57, Lesbian, Lung] (14)
There was no sign of [HCP] discomfort or not knowing how to handle it. I felt at ease being there as his same sex partner. And they respected our relationship and didn’t have any issues whatsoever. [Nathan, Partner, 50, Gay, Head/Neck] (15)
**Egalitarian Practitioner**
**“I treat everyone the same and am not biased in any way”: HCPs Self-Position as Egalitarian Practitioners**
I don’t tailor the care, because I don’t want to be like, oh, you’re a lesbian couple, come here and I’ll do all this fancy stuff with you. I guess I try to treat everyone the same” [Naomi, Allied, 28, Straight] (16)
I don’t take a lot of time to say, ‘so is your partner male or female?’ I don’t know whether or not that’s an important thing to do. I think that people that want to tell you and feel comfortable with you will tell you. [Belinda, Med, 44, Lesbian] (17)
I would be asking about some of these social networks, you know, who’s in their life? Do you have a partner? That’s very often how they’re going to get through. It becomes very much patient led, so the patient can tell you about whatever they want to, if they wish, or not, disclose or whatever. [Brett, Med, 37, Gay] (18)
I let them lead the conversation a little bit. I’d have it if they were prepared to. I don’t have any problems talking about anything that anybody wants to talk about, but probably my confidence in initiating those conversations would be low because I don’t know enough about it. [Jessica, Nurse, 38, Straight] (19)
**Risky Disclosure or Invisibility: Patient Perspectives on Egalitarian Practitioners**
I think it’s really the vibes that they give off. You can’t really pin it to one sort of thing. I think if they’re sort of open, if they’re seeming open and interested in how your life is then that’s a bit of an opening and then you explore a bit and sort of see how they react to other sort of lifestyle things. [Aaron, 32, Gay, Bowel] (20)
You’re constantly having to decide whether it’s worth disclosing to this person, and whether that cost-benefit ratio of how much privacy you have to give up for your care is actually going to pay off. [Dylan, 32, Gay, Non-binary, Leukaemia] (21)
It’s nobody’s business what I do in my private life. I would have to have an enormous amount of trust in them. So, I won’t share none of it with no one. Of course, they are not stupid, they can guess all they want. [Survey, 67, Gay, Head/Neck] (22)
There wasn’t anything specific to same gender couples. There might have been one page out of the whole resource, out of the whole collection of resources. [Cameron, Partner, 38, Queer, Non-binary, Breast] (23)
**Anti-inclusive or exclusionary practice**
**Righteous in Hostility: HCP Engagement in Anti-inclusive Practice**
I’ve heard nurses say, well, I’m entitled to my beliefs that homosexuality is wrong. [Amy, Nurse, 55, Lesbian] (24)
There’s no way anyone is going to openly discriminate or be openly prejudiced. And so those acts are a lot more insidious and subtle. The clinicians will say, oh, no, we do everything great, when in fact they don’t because if you ask the patients, they’ll say, no, they don’t. [Jodi, Allied, 39, Lesbian] (25)
**“You are at their mercy”: LGBTQI Patients and Carers Navigate Anti-inclusive Cancer Care**
I thought that maybe he was just having an off day. But it turns out it wasn’t, he was just a homophobic jerk. He clearly read me as a lesbian and he was dismissive of me as a person, It felt like I was being treated like a lesser person. And that judgment was based on his belief system. [Jasper, 50, Queer, Breast] (26)
That kind of discrimination that is just so constant and covert and daily that it gradually chips away at your confidence and sense of self-worth. [Jessie, 37, Queer, Non-binary/Gender-fluid, Medical Intervention] (27)
At times it felt like medical professionals were reluctant to provide me with any information, and treated me lesser than because I was not a heterosexual white individual. [Survey, Carer, 40, Queer, Non-binary/gender-fluid, Bowel] (28)
You just get that look or that raised eyebrow, or you don’t get referred to properly. I reckon about two out of every ten professionals that we’ve had to deal with, have been a little bit uncomfortable or a little bit weird about it. [Barry, Partner, 56, Gay, Lung] (29)
A couple of times doctors have questioned whether my partner has other family even though I am listed as next of kin on the paperwork. I have found this to be a bit insensitive and it feels like they are looking for more legitimate people to engage with. [Survey, Partner, 39, Lesbian, Brain] (30)
I had difficulty engaging various healthcare professionals because of my presentation as non-binary/trans. I often felt mainstream services did not willingly provide me with the support I needed. So I chose to present as female and made a point to shave off facial hair and present as more feminine. [Survey, Carer, 40, Queer, Non-binary/Gender-fluid, Bowel] (31)
I suspect that the underlying issue with why I would be mis-diagnosed with anxiety might just be that people think gay people are overdramatic or maybe hypochondriacs or something. I certainly wasn’t taken seriously. [Noah, 44, Gay, Lymphoma] (32)
I’ve had operations where I’ve had no pain relief afterwards because the nurse doesn’t like trans people. When you’re on over the night shift and she’s locked your mobile phone in the safe so you can’t call anyone and denied you your drugs. I mean, that’s what we’re talking about with abuse and that’s what bad treatments like and that’s what having someone with you stops. [Scott, 55, Trans man, Gay, Multiple] (33)

# numbers are linked to short quotes cited in the results.

### 3.1 Inclusive and Reflective Practitioner

#### 3.1.1 “I am Proactive in LGBTQI Inclusivity”: HCPs Practice Cultural Competence, Cultural Safety and Cultural Humility

##### 3.1.1.1 “A Legacy of Trauma”: HCPs Acknowledge the Potential Vulnerabilities of LGBTQI Patients

HCPs who adopted a position of inclusive and reflective practice demonstrated cultural competence, proactively creating a place of cultural safety for LGBTQI patients and their partners through practicing cultural humility. The starting point was HCP awareness of the need to “differentiate between people depending on what their sexuality or their gender is” and being open to “change the way we care for people” [Izzie, Allied, 28, Straight], based on this knowledge. Cultural humility also involved HCPs acknowledging the potential vulnerability of LGBTQI people, who may need “more care” because of “extra mental health factors” resulting from “societal discrimination” and potential difficulties in “coming out to family”, or the “potential trauma involved in transitioning” [Amy, Nurse, 55, Lesbian]. HCP recognition of the “legacy of trauma” within healthcare contexts was also evident. This included a “legacy of fear” that LGBTQI identity is “not going to be recognized” or that partners will be excluded by HCPs. As a medical HCP commented:

There’s a legacy of fear within the queer community that you, as a queer person and your queer partner in a queer relationship, are not going to be recognized, and so if something takes a turn for the worse, in terms of somebody’s health, that the partner will be locked out of the room because they’re not officially married, and people fear that. [Suzanne, Med, 40, Queer]

Services could also be “scary” for “people who are intersex” and have had “medical interventions done to you”, meaning that interactions with HCPs in the context of cancer “could be a triggering thing” [Lexie, Allied, 27, Straight].

Inclusive and reflective practitioners recognized “barriers” to patient disclosure of LGBTQI identity within a cis-heteronormative healthcare context. For example, it was recognized that individuals “in that vulnerable situation” have to “go through hoops” and be “brave enough to speak up not knowing what the response is going to be” if they chose to disclose their LGBTQI identity, often having to correct the heteronormative “assumptions” of HCPs [Emily, Allied, 54, Lesbian]. For some HCPs, self-reflection as a “queer person”^1^ precipitated this awareness; for others it was from having LGBTQI friends or family, hearing a “talk at a conference”, or “doing my own research”. Interactions with LGBTQI patients could also result in a moment of enlightenment. For example, one HCP said, “it just really hit me, that it shouldn’t be this way” when a gay male patient “was more worried what I would think about him having a same-sex partner, than actually that he had cancer” [Patrick, Med, 57, Straight].

##### 3.1.1.2 “Non-Judgmental Communication and Support”: HCPs Respect and Acknowledge of LGBTQI Identities, Relationships and Unique Cancer Support Needs

HCPs who adopted a position of inclusive and reflective practice endeavored to ensure LGBTQI patients and their partners felt “welcomed”^2^ and “safe”^3^ by facilitating supportive healthcare interactions within a model of person centred care. Actively demonstrating openness, understanding, respect and acceptance of LGBTQI identities and relationships without a sense of superiority were key attributes of this practice. This reflected an understanding among HCPs that “it’s actually more important to understand and respect who [patients] are before we start telling them what they should or shouldn’t do” [Ayomi, Med, 35, Straight]. Central to inclusive practice was respect for “people’s terminology and self-identification” as well as “really respecting and making their relationship or relationships visible”^4^. Understanding and respect were central to “collaborative decision-making and engagement of our patients”, manifested through “non-judgmental communication and support” and “meeting people where they are at”, rather than “pigeon-holing them in a certain place” [Paula, Program manager, 59, Lesbian].

Sensitivity to the unique meaning of treatment-induced changes for LGBTQI people was part of inclusive practice. This might include the impact of hair loss on “somebody who is trans and they’ve grown out their hair”^5^, “weight loss or weight gain on body image and identity for gay men”^6^, the need for information about the “resumption of anal sex after prostate radiotherapy”^7^, or safe sex information that “doesn’t focus on using a condom”^8^ for lesbian couples. Absence of support networks of some LGBTQI people was also acknowledged: “when you’re looking at an age group of gay men in their 50s and 60s. Life has been difficult for them. Often, they don’t have any family support. They rely on friends for their support” [Cindy, Nurse, 58, Straight]. However, at the same time, not “making assumptions” about the impact of cancer in an individual patient and a willingness to discuss patient needs and concerns were central to inclusive care.

It depends on the individual. If it’s important to them, I think it’s important that they know it’s a platform they are welcome to talk about it. And that’s why I think we need to have an environment that’s very welcoming, but not assume that everybody wants to declare everything all the time. [Melanie, Nurse, 50, Straight]

A cornerstone of inclusive and reflective practice was acknowledgement that it was the responsibility of HCPs to facilitate LGBTQI identity disclosure actively at first meetings with patients, through avoiding cis-heteronormative language and assumptions, “bringing things up proactively” [Russell, Med, 42, Gay]. This could be done through asking questions about “support networks” and “who somebody has in their life, who’s going to support them through their cancer diagnosis”^9^. This then gives the HCP the opportunity to acknowledge the patient’s SOGI status.

I will ask exploring questions around a partner, who’s caring for you, who’s around, who are your supports. And often that sort of open question, to just invite them to describe a bit more, enables them to say, “well, it’s my partner and she is”. And that sort of gives me the opportunity then to acknowledge that they’re same sex attracted [Alison, Allied, 66, Straight].

Directly addressing the question of gender identity by saying to a patient “I’m just going to ask you a few questions about sexuality and gender. And I was just wondering, you know, how do you identify?” can also serve to “provide the space” to allow people to “make the decision themselves as to how much they want to share with you” [Brooke, Nurse, 30, Straight] as a trans or non-binary person.

Respectful reflective practice includes recognition that it is important “to be aware that sometimes the name in the medical notes is not the preferred name” of trans and non-binary patients. This meant it was important “to be very sure, to never dead name them”^10^ [use the name given at birth, before gender affirmation]. If a HCP is unsure about terminology, the identity label, or the name a patient prefers to use, the solution was to “ask them” rather than worrying about “getting pronouns correct”. Avoiding incorrect heteronormative assumptions about the support person of a patient is also important. One HCP said “over the years I’ve found you can make judgments thinking it’s a brother, but it’s a partner or even a father” and thus the solution was to “ask them who they have come with today and often they’ll say, oh, this is my partner. We’ve been together X number of years” [Cindy, Nurse, 58, Straight].

Many HCPs recognized the positive impact of affirmative and inclusive practice on patients in a context where they might be expecting to experience prejudice or discrimination. One medical practitioner who welcomed a male patient’s male partner said, “it was almost like a wall of ice just broke. He [the patient] actually became teary almost of, like of relief” [Patrick, Med, 57, Straight]. A lesbian patient’s wife was described as initially “very defensive of their relationship and her place of next of kin” because of “backlash” at a previous religious hospital, but became “much calmer” once she was “aware that we [the HCPs] took her position as the patient’s partner and main support person seriously” [Survey, Nurse, 27, Straight]. Affirmative and reflective practitioners spent the time “establishing a relationship and letting them [LGBTQI patients] feel that they can talk to you if they want to”, knowing that “over time they tell you all sorts of things” if they feel “safe” [Cindy, Nurse, 58, Straight].

Most HCPs who adopted a position of inclusive and reflective practitioners accepted that it was the responsibility of HCPs to “do the work” to understand the evolving language and terms associated with SOGI identities, and “sit with” their own “discomfort”, if they were unsure about how to interact with LGBTQI patients, reflecting cultural safety and humility.

I think it should sit with us to do our own work to understand the history, to stay abreast of all the evolving language and terms. I think the discomfort as clinicians, we have to be the ones to sit with that. It should not be patients or their families who are feeling like they can’t either disclose important information for their care. And I think as individuals we need to figure out how we can provide better care and more equitable care across all of our patients and keep learning and pushing those agendas through our own teams and organizations [Brooke, Nurse, 30, Straight]

Inclusive and reflective HCPs acknowledged gaps in their own knowledge and confidence, with many commenting on the need for training and communication in addressing the needs of trans and intersex patients. For example, HCPs told us: “say you were a medical practitioner who had no idea what it was to be intersex or trans or non-binary gender, it’s really essential to do your own research and communicate with the patient around about exactly what their own goals and their beliefs and their values are” [Suzanne, Med, 40, Queer]; and “it’s even harder for somebody who’s gender nonconforming or trans to navigate the health care system. I didn’t really deal with that. I’m trying to, trying to learn and do better” [Emily, Allied, 54, Lesbian]. Lack of “support from the top” [Allison, Allied, 66, Straight] for HCP training and education on LGBTQI inclusivity made this process difficult: “We’re hungry for knowledge, I think we have the capacity. We just don’t know where to channel that capacity, and it would be nice to come from a place that’s official” [Amelia, Nurse, 34, Lesbian].

It was recognized that it was the responsibility of those designing healthcare settings and services to provide visible signifiers of inclusivity, such as rainbow flags, stickers and posters in waiting rooms, specific information for LGBTQI people on websites, and identification of gender diversity and sexual orientation on intake forms. This would be “comforting” and indicate “this is a safe space”, making a “big difference for the [LGBTQI] communities” for whom the healthcare setting is potentially “daunting”^11^. However, the majority of inclusive and reflective HCPs described visible signifiers of LGBTQI inclusion in their workplace as an ideal that they would like to aspire to, or “something we could do” to indicate “we respect and celebrate gender diverse individuals here”^12^, rather than the practice at their current place of work. Systemic barriers to signs of inclusivity included difficulties in “accessing the [LGBTQI] material … and we have to get approval from further up the line to do these sort of things” [Cindy, Nurse, 58, Straight]. Others identified the need for education of management and colleagues about LGBTQI inclusivity. For example, one HCP demonstrated agency in adding gender diversity and sexual orientation to patient registration forms and “that was promptly taken off because we had a few patient complaints”. She reflected that “in retrospect, I should have done a bit of teaching and said, ‘right, this is why we’re putting this on there’” [Naomi, Allied, 28, Straight]. Financial barriers in introducing “new models of care” were also identified: “I have to have the business-y, the budget-y hat on. How will this save money. Instead of spending more money. Because we are constrained by that” [Deborah, Nurse, 36, Straight]. These accounts indicate acknowledgement of the institutional barriers to provision of affirmative and inclusive cancer care.

#### 3.1.2 LGBTQI Patient and Carer Perspectives on Inclusive and Reflective Practice: Reassurance and Satisfaction With Health Care

##### 3.1.2.1 “I Knew I Was in Good Hands”: Patients and Carers Feel Safe and Able to Disclose LGBTQI Identity

LGBTQI patients and their carers described interactions with HCPs who adopted a position of inclusive and reflective practice as having direct and positive consequences. Visible signifiers of LGBTQI inclusivity, such as rainbow flags, provided “reassurance” that patients were “going to a safe space”, with “correct values” because of “knowing that the hospital you’re going to is going to be nonjudgmental and treat you as anybody else” [Nathan, Partner, 50, Gay, Head/Neck]. HCPs who were clearly comfortable working with LGBTQI patients served to facilitate feelings of safety, as a carer told us, “I was very lucky to have an accepting environment, especially in the aspects of [HCPs] being comfortable and making me feel safe” [Survey, Daughter, 20, Queer, Adrenal]. Interactions with HCPs who openly identified as part of the LGBTQI community were highly valued in relation to feelings of safety: “out medical staff made me feel safe”; “My GP is a lesbian. I feel very safe”.

Feeling safe meant that patients were confident in disclosure of LGBTQI identity, in the knowledge that they would be accepted without judgement, “my sexuality has mainly been treated as a non-issue. My GP is a gay man so it is openly discussed”^12^; “medical staff never judged my gayness”. Patients and carers commended HCPs who avoided heteronormative assumptions when asking questions, “my experience with the medical practitioners has been positive and inclusive. They have not presumed my sexuality and have asked open questions” [Survey, 43, Gay, Leukaemia]. A lesbian patient with lymphatic cancer praised HCPs who “interacted positively with my children, with my partner and with me”^13^, through asking her children what did they call their two mothers. Sensitivity of HCPs to LGBTQI patients’ fear of discrimination, as well as confidentiality in response to disclosure of identity, was also valued.

My medical team knew that I was transgender and that I feared discrimination. They were very supportive and went an extra step to reassure me. My status as a trans female remained as knowledge with only those that it impacted in my treatment [Survey, 68, Straight, Transgender Female, Head/Neck].

Many patients and carers positioned geographical location as a factor in instilling confidence that they would receive affirmative and inclusive healthcare. For example, participants told us, “I think living and being treated in the inner city means you can take a fair punt on disclosing to health professionals” [Survey, 75, Lesbian, Breast] and “I might not be as accepted as a lesbian in different parts of Sydney and in regional, rural or remote areas of Australia” [Survey, 55, Lesbian, Head/Neck]. Conversely, others valued living in a “rural, small-town area where everyone knows everyone” and which contributed to “being respected”^14^.

##### 3.1.2.2 “There Was Never an Eyebrow Raised”: Partner and Chosen Family Included and Satisfied With Cancer Care

Partner and chosen family inclusion in decision-making processes and day-to-day interactions with HCPs was an important consequence of feeling safe and being able to disclose identity in an accepting and inclusive health care environment. Many patients introduced their partner at a first meeting, “I was deliberately out to my nurses and doctor who’s a world expert. They handled it well, acknowledged my husband, and we use joint decision-making” [Survey, 63, Gay, Prostate]. A lesbian patient said, “I had no trouble at all. My girlfriend participated in meetings and there was never an eyebrow raised or any exclusionary gestures made towards me or her” [Rita, Patient, 61, Lesbian, Cervical). Many partners reported that there was “no sign of [HCP] discomfort or not knowing how to handle it”, which meant that they “felt at ease being there as his same-sex partner”^15^.

HCPs who went beyond non-discriminatory practice in demonstrating cultural safety were highly valued by patients and their carers. One participant said that HCPs “embraced family irrespective of make-up of family”. The partner of a gay man said his husband’s GP had “no issue with (us) going in the consulting room together” and “were just so excited when we got married” [Anthony, Partner, 65, Gay, Prostate]. A lesbian participant described the warmth of HCPs towards her wife:

I usually introduce [wife’s name] as my wife, and we haven’t had anyone flinch or look twice or nothing. We’ve both been included in everything, so they’ll just call us in and just take both our hands on every occasion. Last time when we left the oncologist because my results were really promising he grabbed both of us and gave us a big hug and said ‘you are such a good team’ [Martha, 48, Lesbian, Bowel].

Being able to disclose LGBTQI identity and include partners and other chosen family without meeting prejudice or judgement was also associated with satisfaction with health care, with HCPs described as “brilliant”, “fantastic”, “excellent”, or “great”. For example, participants told us: “All the nurses knew. And all of them were great”; “My own GP is absolutely brilliant … very caring, nonjudgmental and he’s been very good”. Satisfaction was also linked to HCPs being “respectful”, a key attribute of inclusive care. As the trans intersex partner of a woman with breast cancer told us:

As far as the medical people have been with us, we had zero issues. They have always been respectful, and I would always go to an appointment … everyone in the hospitals, doctor’s surgery was brilliant. Surprisingly brilliant. There was never a problem [Kai, Partner, 50s, Bisexual, Trans, Intersex, Breast].

Others said “all of the medical staff involved treated me with respect. They also treated my wife with respect [and] I felt acknowledged and respected as a partner and carer” [Survey, 69, Lesbian, Endometrial]. HCPs acknowledging LGBTQI status, while treating the patient “as a person” was manifestation of this respect, “he just treats the person as a person he doesn’t go, ‘Oh well, I’m going to have to put a label on you now because you told me that you’re bisexual’” [Grace, 56, Bisexual, Cervical]. In combination, this resulted in the positioning of HCPs as “really fantastic in terms of communicating (and) supporting” [Ruby, Partner, 60, Lesbian, Bowel], “really great”, “really good”, and as “exceptional”.

### 3.2 Egalitarian Practitioner

#### 3.2.1 “I Treat Everyone the Same and Do Not Discriminate”: HCPs Self-Position as Egalitarian Practitioners

##### 3.2.1.1 “Cancer Is the Same for Everyone”: HCPs Don’t Need to Do Anything Different for LGBTQI Patients

HCPs who adopted the subject position of egalitarian practitioners reported that they treated “everyone the same”, regardless of gender and sexuality. Many HCPs stated that cancer was the same for everyone and “I don’t see that there’s a huge difference in the care of the cancer itself” [Omar, Med, 60, Straight], hence “I don’t think there’s a need to do anything different” for LGBTQI people [Patrick, Med, 57, Straight]. As long as patients were “getting good care for their cancers”, organizations were believed to be “doing enough”, with “other problems identified” being “referred to psychological services”, implicitly pathologizing LGBTQI identities [Kylie, Nurse, 60, Straight]. LGBTQI patients were considered to be no different from any other cancer patient in facing “concerns about survival and the concerns of recurrence of disease”, or in palliative care, “the same end of life physical, emotional and psychosocial issues”.

As a gastroenterologist I don’t think it’s that important. For treating cancer, so you’re talking about people coming in for chemotherapy, sitting for hours, feeling sick. I think there it might be important to have something visual for them … that you are welcome here. I don’t think in my context there’s necessarily a need to do anything different … we don’t have anything special for them [Patrick, Med, 57, Straight].

HCPs told us that information about “safe sex in regards to treatment … doesn’t need to be any different for a gay or straight person” [Darren, Allied, 53, Gay] and hence “I try to treat everyone the same”^16^. Some HCPs positioned others as responsible for affirmative and inclusive care, arguing that there was no need for acknowledgment of LGBTQI status in “frontline care work”, because “support services probably are doing all that stuff” [Melanie, Nurse, 50, Straight]. Others made a distinction between cancers of the reproductive organs, such as prostate and breast cancer that “might affect their identity” and cancers such as lung, gastrointestinal and bowel cancer “where the effect is the same. It’s kind of fairly similar regardless of your gender or your sexuality” [Ayomi, Med, 35, Straight]. Many egalitarian HCPs reported “comfort and confidence” in providing cancer care for LGBTQI patients even though they had not “looked outside for training or things that exist that could help my knowledge” [Cristina, Allied, 35, Straight].

Egalitarian HCPs believed that there was no need to “display anything” that was explicitly LGBTQI inclusive, such as “wear a rainbow lanyard”, or “do anything that says I’m one of the people you’re welcome to talk to” [Melanie, Nurse, 50, Straight] because they were **“**friendly to everyone” [Ken, Med, 50, Straight]. Drawing on discourses of ethical responsibility, these HCPs said that all patients were “given the same respect and care, no matter race colour or sexual outlook” [Valentina, Nurse, 56, Straight] and LGBTQI patients were treated “how I would treat every other patient” [Kylie, Nurse, 60, Straight]. A number of HCPs who adopted the position of egalitarian practitioner stated that they were unsure why there was a need to “single out a particular population” as “surely we are well past that”, and “if you start being too demonstrative being LGBT friendly, it almost … draws particular attention to it” [Brett, Med, 37, Gay]. Many HCPs positioned themselves as “inclusive” and non-discriminatory because they treat everyone equally, providing the same “high quality” service to “anyone who needs it [Cristina, Allied, 35, Straight].

I do think that we are quite inclusive and we don’t discriminate. Therefore … we’re treating everyone equally, and I think that’s what it should be about, is everyone getting equally good support [Darren, Allied, 53, Gay].

From this standpoint, LGBTQI identity disclosure was positioned as irrelevant to the provision of patient care, including disclosure of sexuality, gender identity and intersex status. This draws on a discourse of equality, suggesting that everyone is treated the same, rather than equity, whereby everyone is provided with what they need for good healthcare provision.

##### “Patients Will Tell Me If They Want to”: HCPs Do Not Facilitate LGBTQI Identity Disclosure

HCPs who adopted the position of egalitarian practitioner did not explicitly facilitate disclosure of LGBTQI status as it was assumed that “people that want to tell you and feel comfortable with you will tell you”^17^. As a result, disclosure was “very much patient-led”. Some healthcare professionals did use neutral language to ask about “social networks”, such as, “who’s in their life?” or “do you have a partner?”^18^ if they “sensed” or “picked up” that the patient may be LGBQTI, suggesting awareness of the importance of inclusivity. However, it was acknowledged that “if they didn’t have a partner then maybe it wouldn’t come up. It doesn’t get asked at all” [Amelia, Nurse, 35, Lesbian]. Patients who did not appear to the HCP to be LGBTQI would also be overlooked as the HCP would not adopt neutral language. Equally, identification of a person as trans, non-binary or having an intersex variation would not follow on from questions about social networks or partners, resulting in HCPs “missing people”.

I don’t tend to ask people. I don’t proactively ask people do you identify as LGBTIQ. I sort of pick up on it if it’s there. But, you know, that probably means that even I am missing people. Sometimes, I’ve been in a situation where I’ve had a trans patient, for example, and they just really pass. I’ve only realized that they are trans when I do a physical exam [Suzanne, Med, 40, Queer].

This HCP did demonstrate some reflectivity, commenting, “it’s maybe something that I could improve on in my own practice”, but explained “it’s not sort of something that’s taught to us”.

##### 3.2.1.3 “My Capacity to Actually Get It Wrong Is Massive”: HCPs Are Out of Their Comfort Zone and Don’t Want to Cause Offence

A number of HCPs accounted for the fact that they did not ask about LGBTQI status, or actively facilitate disclosure, by stating that they did not want to “make assumptions” due to the fear they would be seen to be “overstepping” or “going down a track that could be offensive” to non-LGBTQI patients [Darren, Allied, 53, Gay]. It was also argued that some “people that did identify [as LGBTQI] might think ‘it’s none of your business’” or might experience the HCP as voyeuristically “gaping” at them, or respond negatively to uninformed or “insensitive” HCP questions. Other HCPs were concerned about displaying LGBTQI inclusive signage because of concern “it would antagonize one or more of my conservative patients” [Lynette, Med, 58, Lesbian] and “there are still a lot of people out there who are not comfortable with gay and lesbian couples” [Patrick, Med, 57, Straight]. As a result, HCP participants said, “it’s probably better to stay neutral” and let “patients … identify to you” or “lead the conversation”^19^.

After a patient’s disclosure, a number of HCPs were concerned that they “would offend somebody because of my lack of information” [Katrina, Allied, 64 Straight] or were “worried about calling them the appropriate term”, “which could serve to “take away from them just being my patient and treating them well” [Survey, Nurse, 48, Straight]. More specifically, lack of knowledge and confidence in “language to do with transgender people” was described as making a number of HCPs feel “inadequate and probably a little bit embarrassed” or “nervous and cautious” [Kelly, Nurse, 60, Straight]. This was because of a fear that their “capacity to actually get it wrong is massive”, which could “cause offence or damage rapport” [Leanne, Allied, 47, Straight]. As a result of this “fear of stepping on toes with fear of being offensive”, many HCPs simply said “nothing at all”, which was acknowledged by some to be “not very good either” [Alia, Allied, 31, Straight].

#### 3.2.2 Risky Disclosure or Invisibility: Patient and Carer Perspectives on Egalitarian Practitioners

##### 3.2.2.1 “Another Layer of Things to Worry About”: The Emotional Work of LGBTQI Identity Disclosure

HCPs who adopted a position of egalitarian practitioner and did not “open up” the discussion of SOGI status, were seen by patients and their carers as assuming a patient was “straight” and cisgender, and that their partner was “a friend”. This was a source of dissatisfaction with healthcare, which LGBTQI participants said, “really pisses me off” and “creates a lot of stress”, because “if they didn’t make that assumption automatically that I was heterosexual then I think it would have been a lot easier to handle” [Christine, 53, Lesbian, Ovarian and Uterine]. Failure to acknowledge gender diversity was also a concern for many patients.

What I would have liked them to do was to ask me what pronouns I would like. Would I like to be called ‘he’ or ‘him’ or ‘she’ and ‘her’ or ‘they’ and ‘them’. They didn’t ask [Lauren, 63, Queer, Trans, Prostate]

Egalitarian practice puts the onus on patients and their carers to disclose in a context where they are unsure about the response they will receive from HCPs. As one participant reported, “having to explain every time that you are not straight was another layer of things to worry about or have to deal with. I already had enough going on just with the treatment” [Survey, 56, Lesbian, Breast]. The “anxiety around disclosure” and repeated decision-making before an encounter with a new HCP about “when do I bring it up, how do I bring it up?” [Dylan, 32, Gay, Non-binary, Leukemia] was described as “emotionally extremely draining” [Scott, 55, Gay, Trans man, Multiple] and “a little bit wearing after a while” [Paulette, 67, Lesbian, Colorectal]. LGBTQI patients and their carers were thus “on a merry-go-round” of “outing yourself the whole time” as well as “outing your partner if they’re with you”. This meant they were leaving themselves open to HCPs “not being too receptive”, fearing that HCPs will “change their mindset and how they treat you” after disclosure. This was “emotional effort” and a “burden” on LGBTQI patients [Paulette, 67, Lesbian, Colorectal].

Being part of a marginalized community brings additional pressures and stresses, and the anticipation of potential discrimination, or everyday misunderstanding, is always there. This creates additional burdens which impact on health and wellbeing. This awareness needs to be out there [Survey, 52, Lesbian, Breast].

There were a number of ways LGBTQI patients and carers responded to their uncertainty about HCP responses to disclosure. Some individuals would assess “the vibes they [HCPs] give off”^20^ at a first meeting, or “call the doctor’s office and tell them in advance so I can gauge their reaction before I go in”. Selective disclosure on a “needs basis” was also reported, only happening if the patient “considered it relevant” to their care or felt confident in a positive HCP response. Others, most commonly older cisgender gay and lesbian cisgender individuals, said that they were “always open and honest with our providers”, and because I am “out and comfortable with who I am” or “proud of who I am. I don’t hide any more”, expecting “others to treat me accordingly, especially around such an emotional and fraught issue as cancer” [Survey, Carer, 77, Lesbian, Ovarian] and include their partner in all discussions. Some self-proclaimed “very out” participants reported a more “assertive” response, refusing to “tolerate any kind of homophobic bullshit”, or saying, “if you don’t like who I am, I don’t care, you’re shit” [Rita, 50, Lesbian, Cervical].

If HCPs who adopted a position of egalitarian practitioner responded positively to LGBTQI disclosure, this had positive consequences in terms of patient satisfaction, engagement with care and inclusion of partners, as reported in interactions with inclusive practitioners. For example, a carer of her partner with ovarian cancer, said she was “always wary wherever I am … judging it all the time so that I can act appropriately to be safe”, but “not once did I feel a lesser person or was judged”, even though “a few of the health care professionals might have made a mistake and thought that we were sisters”. She drew on a metaphor of horse training to describe how she interacted with HCPs:

I used to breed horses and train and break horses, so we had this joke that I always had someone else to break in. But we do it very well. And I think it is very helpful in how they [HCPs] treat you. You know, they’re humans and they lack knowledge as well. It’s a two-way street. But I didn’t feel any homophobic times through all of [partner’s name]’s treatment, which I think is just amazing. It just goes to show how far we’ve come [Claire, Carer, 66, Lesbian, Ovarian].

However, many other LGBTQI patients and carers reported feeling “judged”, or positioned as a “weirdo” or as a “Martian” following SOGI disclosure in interactions with HCPs who were well meaning but “needed more education on inclusivity and how to discuss these topics without being offensive” [Survey, Partner, 20, Queer, Non-binary/Gender-fluid, Breast]. For example, a non-binary participant reported feeling like a “fascinating test subject” whose use was in educating HCPs, while paying for the privilege through private health care.

I find it really hard to even transfer between medical professionals because people want to hold on to me cause I’m like a valuable patient to have on their books. There was one health practitioner last year … I just felt like she was ripping me off and just finding me really fascinating, like I was like educating her and then paying for it at the same time. [Jessie, 37, Queer, Non-binary/Gender-fluid, Medical intervention, multiple cancers]

Some participants dealt with visible HCP discomfort or lack of knowledge calmly by being “personable and engaging” and assuming HCPs would accept them: “I’ve never made being gay a ‘problem’ and if there was a ‘problem’. I have always approached its resolution in a caring open way” [Survey, 67, Gay, Prostate]. Others reported feeling “a bit uncomfortable” because of the obvious “discomfort” of HCPs following disclosure, or felt it was “insulting and insensitive” to have the impact of cancer dismissed after they disclosed.

I had two people (HCPs) say, ‘it doesn’t matter, you’re a lesbian’. And I said, ‘I don’t understand what you mean, why does it not matter that I’ve got cancer because I’m a lesbian?’ And after the blushing, they go ‘well you’re not having [penetrative] sex’… There was an assumption that it’s okay to have breast cancer if you’re lesbian because a lover will understand your situation or your lack of sex drive, and it won’t matter because you’re not with a bloke. [Myra, 68, Lesbian, Breast]

Lack of HCP awareness of the intersection of cultural identity and LGBTQI identity was also commented upon. For example, a participant from a Chinese cultural background said, “few [HCPs] consider the points of differentiation for lesbians from culturally and linguistically different backgrounds” [Violet, 53, Lesbian, Uterine], and an Aboriginal man told us, “there’s a lot of complexity around the intersection of sexuality and cultural background and race, and health care settings in Australia are not geared towards acceptance around that” [Ryan, 60, Gay, Prostate]. HCP assumptions based on the cultural background of the patient were sometimes incorrect: “I’m not out to my parents and there were a lot of cultural assumptions. Being Chinese the doctors were assuming that my parents should be involved in the decision making, whether or not I wanted them to be involved” [Ash, 40, Non-binary, Bisexual, Unknown cancer].

##### “They Never Ask; I Never Tell”: Non-Disclosure Means Safety and Privacy, as Well as Invisibility and Unmet Needs

Many LGBTQI patients and carers dealt with uncertainty about HCP responses to disclosure by choosing not to disclose their SOGI status. As one participant told us, “Doctors? They never ask; I never tell” [Survey, 69, Queer, Prostate]. Non-disclosure had both positive and negative consequences. Some participants described concealment of LGBTQI status as “easier” and “safer” because the “cost-benefit”^21^ analysis of coming out resulted in feelings of “trepidation”, with disclosure positioned as “too scary” and “even opening this conversation” as “often-impossible”. It was believed that “in not being out, you get treated better”, with some participants describing a sense of agency in “determin[ing] when and how others know”, thereby allowing them to avoid discrimination.

I always tick women on the forms because it’s so discriminatory if I don’t. It is just absolutely not worth it to me to identify as anything other than cis in the health system because people make a mockery of trans bodies. I ride off the privilege of my gender fluidity constantly in order to grin and bear it, deal with the cis-normativity that it takes to avoid that aspect of discrimination. [Jessie, 37, Queer, Non-binary/Gender-fluid, Medical Intervention, multiple cancers]

Ticking “woman on the forms” was not without cost, however, with Jessie saying “I had to sacrifice that part of my identity to get treatment in the health system”. This had negative implications for their health, as they had “come to points in my life where I’ve avoided help seeking or opted out of the health system just because I couldn’t be a binary person that day”.

For others, LGBTQI status was deemed “irrelevant” or “not necessary to declare” in relation to cancer care as it was “nobody’s business what I do in my private life”^22^. However, non-disclosure meant that cis-heteronormative assumptions remained unchallenged, which could leave individuals feeling “awkward and uncomfortable”, “silenced”, “angry”, “guilty” and “not understood”, because their LGBTQI status was erased or made invisible by HCPs.

Frustration was common when requests for LGBTQI specific information were ignored, or “general information” provided in response to requests. For example, the response to a gay man who asked for information about “what to look out for” when having sex after treatment was “a verbal off the cuff ‘practice safe sex’, in general terms” [Carter, 21, Gay, Leukemia]. The “absence of targeted information” and support to address LGBTQI patient needs reinforced feelings of invisibility as “there wasn’t anything specific to same-gender couples”^23^, or for “trans and non-binary bodies” available for most participants. As one carer commented, “It’s really difficult to find support [online or face-to-face groups] that include lesbian women. My partner had a gynecological cancer, so all the supports were aimed at male partners” [Survey, Partner, 54, Lesbian, Ovarian]. Another said, “there are resources for carers and resources for individuals with cancer, what is lacking are services who understand the complexities when you add LGBTQI+ into the mix” [Survey, Parent, 40, Queer, Non-binary/Gender-fluid, Colorectal]. This led to many patients and carers feeling “despondent” and “isolated by mainstream cancer supports”.

### 3.3 Anti-Inclusive Practitioner

#### 3.3.1 Righteous in Hostility Towards LGBTQI People: HCPs Engage in Anti-Inclusive Practice

HCPs who adopted a position of anti-inclusive practice demonstrated negative attitudes or outright hostility toward LGBTQI patients. This was evident in the accounts of a small minority of HCP participants who complained that the “abnormal behaviour” of LGBTQI people was being “forced” onto them and that they “just don’t need to hear about their (patients) sexual orientation if it has nothing to do with treating their condition” [Survey, Nurse, 61, Straight].

I don’t see why everyone has to force their sexual orientation on others. Heterosexual people don’t go around talking about their sexual orientation. I am now forced into hearing about and watching abnormal behavior on TV and more advertisement of non-heterosexuals. [Survey, Nurse, 61, Straight].

More commonly, the anti-inclusive practices of colleagues were observed by other HCP participants. This included accounts of HCPs who were righteous in their exclusion of LGBTQI patients, feeling “entitled” to their beliefs “that homosexuality is wrong”^24^. HCPs were observed to behave in “insulting”, “disgusting” and “unnecessary” ways that “show lack of understanding and lack of respect” for LGBTQI patients. This was particularly acute in relation to trans patients. For example, HCPs described observing “misgendering practices” by “a few of the doctors and some nurses” in an outpatients clinic; or HCPs “intentionally using the wrong pronouns and saying derogatory things” [Amelia, Nurse, 35, Straight] about a trans patient who was attending for an appointment; and behavior described as “an aggressive act” and “micro-aggressions” [Amy, Nurse, 55, Lesbian]. HCPs also reported anti-inclusive practices in the form of “insidious and subtle”^25^ micro-aggressions. This included colleagues “tutt[ing] under their breath” at “the badges around the place saying trans ally”, or providing “lip service” to LGBTQI inclusion, while concealing their “implicit biases” because they were “too clever to be openly discriminate” [Jodi, Allied, 39, Lesbian].

Some HCPs acknowledged that the anti-inclusive practices they observed had material consequences, with cis-heteronormative assumptions about patients resulting in “important pieces of information … missing from that interaction”, which meant that “the patient might not feel safe to ask the questions, clarify or seek support” [Tammy, Nurse, 48, Straight]. One HCP observed the withholding of fertility preservation advice for a man because he was gay:

The consultant looked at me and said, ‘oh, I don’t think that’ll be an issue’. I knew that the consultant was assuming he was gay, but then taking that next step and assuming that he wouldn’t be having children. To me, that wasn’t an appropriate assumption to make [Ayomi, Med, 35, Straight].

It was acknowledged that anti-inclusive comments between colleagues could be damaging because “even if the patient didn’t hear, it’s still encouraging that sort of culture in the workplace” [Amelia, Nurse, 35, Straight].

Many of the HCPs recognized that challenging anti-inclusive practice observed in colleagues was important. HCPs who it was assumed were “well-meaning” and “don’t come from a bad place” were seen to “need a bit of a fact check” about comments that were “really just not appropriate” or “careless”. However, trying to “educate” colleagues “who are prejudiced to LGBTQI patients” and are coming “from a place of harm” [Alia, Allied, 31, Straight] was reported to be more difficult, as negative attitudes and “discrimination” toward LGBTQI people was often “ingrained”. HCPs explained that they “could spend three minutes or three hours here and your mind might never be changed” [Jessica, Nurse, 38, Straight], as “there’s a lot of bigots out there and there’s a lot of bias still in health” [Kelly, Nurse, 68, Straight]. Others explained that prejudicial behaviour on the part of their colleagues that “could go to disciplinary action” was not pursued, in part due to lack of confidence that “upper management would have really recognized the importance” [Amy, Nurse, 55, Lesbian].

#### 3.3.2 “We Are at Their Mercy”: LGBTQI Patients and Carers Need to Navigate Anti-Inclusive Cancer Care

##### 3.3.2.1 “The Biggest Area That I’ve Felt Discriminated in”: The Damaging Impact of Interactions With Hostile and Offensive Health Care Professionals

The impact of anti-inclusive practice on LGBTQI patients and their carers was universally described as negative and damaging. A substantial number of patients and carers concurred, “not everyone in the medical team was accepting or supportive”, providing examples including doctors, nurses and allied health professionals. LGBTQI patients and their carers described having to navigate the “constant”, “covert” and “daily discrimination” in cancer care. Believed to be “everywhere”, anti-inclusive HCPs were described as “positively hostile”, “dismissive”, “paternalistic and judgmental” of LGBTQI patients. This resulted in the feeling of “being treated like a lesser person”^26^ because it can “gradually chip away at your confidence and sense of self-worth”^27^. It was “stressful” to sense a negative “vibe” from an anti-inclusive HCP, suggesting that they “don’t want you here”, leading to feelings of “distrust” towards HCPs.

2011_may_w_g2.ddsIn health, where you are just naked all the time … everything that is intimate and important to me has been clinically invaded by people who don’t respect me for who I am. So those people are everywhere. That systemic discrimination makes me distrust people in the system who do really good work and do really care [Jessie, 37, Queer, Non-binary/Gender-fluid, Medical Intervention].

Some anti-inclusive HCPs were reported to change from being “warm and helpful” to “cold” and “shorter in their responses”, or to have “stopped speaking to me” when patients disclosed their sexual orientation, intersex variation, or trans status:

Two of my specialists stopped speaking to me after my sharing about being intersex. It’s clear there is a great deal of stigma surrounding it [Terry, 40, Queer, Non-Binary, Intersex, Medical Intervention]

Due to my gender presentation, I often felt mainstream services did not willingly engage with me or provide me with the support I needed [Survey, Parent, 40, Queer, Non-binary/Gender-fluid, Colorectal]

I’ve had some that I’ve said I’m gay and they’ve just sort of shut down after” [Aaron, 32, Gay, Bowel].

Some HCPs were overtly exclusionary, stating to patients that they don’t agree with “that sort of thing’”, or that the patient was not “living according to God’s will” because of being gay. One HCP reportedly “dropped her hand and said ‘not in this hospital’ and left” [Myra, 61, Lesbian, Breast] when she realized she was discussing assisted reproduction with a lesbian woman. Patients also told us that their HCP ignored their disclosures of identity, for a participant said; “I had told him that I was a gay woman. He still asked to talk to my husband” leaving her feeling as though “he didn’t see me”, “didn’t hear me”, “didn’t understand who I was” [Barbara, 48, Lesbian, Uterine]. Trans and non-binary patients explained that it could be “difficult” to get HCPs to “use gender-neutral language”, including one young person who “had to beg” their oncologist “to stop mis-gendering me”. These responses to disclosure reinforced “distrust” and a distinct lack of safety. As one participant told us:

I don’t feel safe. I have to think ALL THE TIME in medical situations if it’s safe to come out. Correcting, educating, making formal complaints – I am enraged that my energy has been taken up by this my whole life when I’m in pain; very sick; recovering; scared. [Survey, 39, Queer femme, Medical Intervention].

Offensive comments or actions by HCPs could also be a source of distress for LGBTQI patients. For example, one lesbian participant reported, “a doctor told me I shouldn’t have an issue with her putting her fingers inside of me ‘to test’ something … because ‘people like you like this kind of thing’” [Survey, 40, Lesbian, Cervical]. A bisexual woman who disclosed to her doctor that her fiancé was a woman was asked “do you consider yourself to be a man?”, leading to the reflection “that was another situation where I become the educator instead of being a patient” [Catherine, 61, Bisexual, Vulval]. Anti-inclusive practices were experienced as more all-pervasive for some patients living in regional and rural locations, because HCPs can “get away with having biases and being discriminatory when there are limited options for the patients” [Survey, 63, Straight, Breast]. As a trans participant told us, “If you live in one of the small towns, you don’t get to choose who your GP is. They might be very transphobic and you’re stuck with them” [Victor, 47, Straight, Trans Man, Ovarian].

##### 3.3.2.2 Denigrated or Ignored: Active Exclusion of Partners and Chosen Family Carers

Many partners and other carers reported being impacted upon by anti-inclusive practices, feeling that HCPs were “reluctant” to engage with them, or treated them as “lesser than” because they were not “a heterosexual white individual”^28^. Partners reported, “you just get that look or that raised eyebrow, or you don’t get referred to properly”^29^, with HCPs “insisting on referring to me as his friend” despite “being told we were married”. Another HCP “questioned” whether the patient had “other family”, as though they were “looking for more legitimate people to engage with”^30^. Patients also spoke of partner exclusion:

My radiation oncologist clearly thought my life was absolutely disgusting, refused to acknowledge my partner. If she was in an appointment with me, he’d just completely ignore her. I had ticked the de facto box and he actually scribbled out my tick on that box and put single [Catherine, 41, Lesbian, Vulval].

Another patient told us that it was “difficult for my partner to get any answers and yet when my parents turned up they were more than happy to talk to them” [Survey, 42, Lesbian, Uterine]. Administrative staff, who selectively applied hospital policies, also perpetrated “woeful” exclusionary practices. For example, a lesbian lung cancer patient’s wife and partner of 25 years was required “to stay outside” on the basis that she “wasn’t family yet”, an incident that happened just before marriage equality was legalized in Australia. Hostilities were also extended to chosen family, such as “lesbian friends” who would “come and visit” such as being treated “quite offhandedly”, “eye rolling” and with lack of “respect” [Elsie, 55, Lesbian, Lung]. Intentional refusal to recognize LGBTQI partners had “horrible” consequences for one gay man who, despite having “power of attorney and enduring guardianship” for his partner, found that “the doctor in charge wouldn’t let me see my partner when he was dying because we’re gay”. He concluded “I think the doctor just did not like gay people”, evidenced by broader homophobic assumptions on display:

I felt my partner wasn’t treated with dignity and respect. And I wasn’t treated with any dignity or respect when my partner was dying. They were quite rough, without even warning me. Like he’s from out of space or like he’s got AIDS. Taking it for granted because he’s gay then he’s got AIDS [Neal, 68, Gay, Prostate].

##### 3.3.2.3 “They Don’t Want Me to Live Because I’m Gay”: Fear of Inadequate and Insensitive Cancer Care

Numerous LGBTQI patients reported instances wherein they perceived their medical care to be inadequate, or feared being denied health care services because they were LGBTQI, with direct implications or their willingness to engage in cancer healthcare. As one young lymphoma patient told us, “what if people don’t want to treat me because they don’t want me to live because I’m gay” [Oscar, 27, Gay, Lymphoma]. Issues included, “difficulty engaging” HCPs “because of my presentation as non-binary/trans”^31^; being misdiagnosed due to beliefs that “gay people are overdramatic or hypochondriacs”^32^; “fertility issues” not being discussed “as part of cancer care because I’m gay”; and being denied “pain relief” after an operation “because the nurse doesn’t like trans people”^33^.

When we go into a random appointment, we might be looking at someone who actually wants us dead. That is how hard it is to get medical care. You’ve randomly got to work out a way to protect yourself against someone who really doesn’t know where the problem is and hates your guts [Scott, 55, Gay, Trans man, Multiple].

Patients also reported the distress they experienced following encounters with HCPs who deliberately enforced cis-heteronormative ideals through their clinical decision-making. For example, one HCP was reportedly “focused entirely” on maintaining a lesbian patient’s vagina with dilators post-surgery “so that a man could put his penis in it” if she decided to be in “a proper relationship one day”. This was despite the patient telling him “that was not an issue, he [HCP] would just ignore me, just talk over the top of me” [Catherine, 61, Bisexual, Vulval]. A number of participants reported feeling judged in their choices in relation to reconstruction following breast surgery. A non-binary participant said that they “had to fight really hard to not have a reconstruction after a mastectomy”, and another patient said that there was a “lack of understanding” of LGBTQI patients’ “desire to go flat” [Jasper, 50, Queer, Breast]. A carer told us:

My partners’ surgeon made her feel like a weirdo for the plastic surgery options she requested and didn’t really know how to be neutral on the topic of gender nonconformity and transgender identities with her other patients. She needed more education around how to discuss these topics without being offensive and making us feel like total oddballs for who we are [Survey, Partner, 33, Queer, Breast].

LGBTQI patients and their carers reported detrimental impacts of anti-inclusive and exclusionary care, including feeling as though it “prevents me from help-seeking for my current maintenance care” [Patricia, 65, Lesbian, Uterine]. Although many patients positioned themselves as “assertive” in their lives generally, in the context of cancer care they reported feeling “at the mercy” of their HCPs [Hannah, Partner, 45, Lesbian, Uterine]. A number of patients reported feeling that “you can’t really complain” and that “not seeing that person again” was “not a choice that you get”, as anti-inclusive HCPs may be “the only thing standing between you and death at that point in time. You don’t have the luxury of just walking out” [Catherine, 61, Bisexual, Vulval].

## 4 Discussion

The aim of the present study was to examine the construction and experience of LGBTQI cancer care from the perspective of HCPs, LGBTQI patients and their caregivers. We identified three subject positions adopted by HCPs in relation to the provision of care to LGBTQI people: inclusive and reflective practitioner, egalitarian practitioner, and anti-inclusive practitioner, which had implications for LGBTQI cancer patients and their partners, and other chosen family caregivers.

HCPs who took up the subject position of inclusive and reflective practitioner demonstrated LGBTQI cultural competence and cultural humility, creating a place of cultural safety (33–35) for LGBTQI patients and their carers, through a range of inclusive verbal and non-verbal strategies (1, 14, 45). Inclusive and reflective HCPs regarded LGBTQI patients as potentially vulnerable and needing nuanced care, following best practice models of person-centered care tailored to individual patient needs (66). They recognized the impact of societal discrimination and the legacy of trauma in health care, including difficulties related to disclosure of SOGI status (67) and violations to bodily autonomy for some intersex patients (68), drawing on an affirmative construction of LGBTQI health (69). Inclusive and reflective HCPs acknowledged the need for sensitivity and acceptance of SOGI status in interactions with LGBTQI patients, and the intersection of identities in LGBTQI patient outcomes, including sexuality, gender, age and cultural background, which can lead to discrimination across “multiple axes of oppression” (20). Inclusive HCP practice involved non-judgmental respectful treatment and welcoming and open dialogue, accompanied by reflective awareness of gaps in their own personal knowledge and skills (1, 14, 45). The importance of knowing patients’ SOGI status information was acknowledged (14, 28), and the assumption that all patients are heterosexual and cisgender was avoided, by HCPs taking responsibility to facilitate disclosure of patient SOGI status, and including partners and other chosen family in consultations and care. Inclusive and reflective HCPs recognized the importance of the relationship between clinicians and LGBTQI patients in the provision of affirmative health care (31).

This model of inclusive and reflective practice is an exemplar of communicative competence, identified in previous research on LGBTQI cancer care (1, 6, 14, 28, 45). This practice had direct positive consequences for LGBTQI patients and their carers, in terms of feeling safe and respected in interactions with HCPs, willingness to disclose SOGI status with the knowledge that there would be a positive response, and satisfaction with cancer care, aligned with prior literature (4, 12, 13, 48). Our LGBTQI patients and carers told us that this is what they want in cancer care. Previous research has established that LGBTQI patients who disclose SOGI status in the context of general healthcare, and who receive a positive and accepting response from HCPs, report greater satisfaction with care and increased likelihood of engagement with health screening (9, 13, 70–72). This has direct positive benefits for physical and mental health (72–74). At the same time, inclusive and reflective HCP provision of tailored information in response to individual needs, access to LGBTQI specific support groups if available, and acknowledgment of the need for visible signs of LGBTQI inclusion, serves to address gaps in generic cancer information and support for LGBTQI people and their carers (4, 6, 28). It also ensures that treatment decision-making is informed by LGBTQI patient needs and the potential impact of cancer treatment on identities and relationships (15, 75).

HCPs who adopted the subject position of egalitarian practitioner drew on discourses of ethical responsibility to position themselves as offering an inclusive high-quality service to all patients, a mode of patient-clinician interaction identified in previous research (1, 14, 45). Knowledge of SOGI status was deemed irrelevant in the provision of cancer care for some egalitarian HCPS, or only relevant for patients with cancer affecting sexual or reproductive organs. This reflected a construction of LGBTQI identity as primarily about sexuality (76, 77), negating the importance of acknowledging LGBTQI patient needs in all tumour types (3). It also confirmed previous reports that the majority of oncology HCPs do not inquire about a patient’s sexual orientation when taking a history (1, 2, 14, 38, 41), with many not seeing the relevance of knowing the SOGI status of their patients (1, 37, 45).

Some HCPs who adopted a position of egalitarian practitioner did have knowledge about gender-neutral non-heteronormative language, such as referring to ‘partners’ rather than ‘husband or wife’ and recognised its importance in the provision of inclusive and affirmative cancer care. However, affirmative language was only used in interactions with patients identified by HCPs as LGBTQI, implicitly drawing on stereotypical notions of LGBTQI appearance (78) and overlooking the substantial proportion of LGBTQI people whose SOGI or intersex status is not visibly identifiable to others (79, 80). Self-positioning by some egalitarian HCPs as being uncomfortable, unskilled or lacking in confidence, reflected in concerns about causing offence to non-LGBTQI patients, or use of correct terminology with LGBTQI patients, in particular with transgender patients, has been reported previously (1, 2, 14, 37, 45). This demonstrates lack of awareness of specific strategies of communicative competence needed to care for LGBTQI patients (81), potentially compounded by the many challenges associated with uptake of best practice guidelines (82). It also demonstrates lack of awareness of the negative impact on LGBTQI patients if HCPs do not adopt inclusive and affirmative strategies (4, 9). However, the evidence of self-reflection in these accounts suggests that some practitioners who adopt an egalitarian subject position may be able to move to an inclusive, reflective practitioner position with the right skills, education and support.

There is growing acknowledgement the position of treating all patients the same is an unhelpful mode of practice, described as a ‘micro-aggression’ (45) that serves to minimize health disparities experienced by LGBTQI patients. It also exonerates HCPs from needing to acquire specific knowledge or training in order to care for LGBTQI patients, or need to engage in reflective practice in their clinical interactions (1, 83). Egalitarian practitioners who use the ‘same yardstick’ to address the concerns of their patients are implicitly signaling a cis-heteronormative subject position, which does not acknowledge the unique needs of their LGBTQI patients (1, 14). This is not following guidelines for equitable person-centered care (66), and serves to render LGBTQI patients and their carers invisible (84). Cis-heteronormative assumptions on the part of oncology HCPs and absence of opportunities for SOGI disclosure are associated with LGBTQI patient dissatisfaction with healthcare (4, 12) and anxiety about disclosure of SOGI status (4, 12–14), and this was confirmed by patients and carers in the present study. In the absence of visible indicators that healthcare settings or individual HCPs were inclusive and affirmative in their practice, many LGBTQI patients and their carers feared that they would face HCP hostility and discrimination, and be offered substandard cancer care (4, 12, 13, 47, 48). This added to the psychological burden of dealing with cancer diagnosis and treatment, resulting in feelings of distress and frustration throughout the cancer care process (4, 13).

A minority of HCP participants in the present study adopted the subject position of anti-inclusive practitioner, expressing open hostility and prejudice toward LGBTQI patients. Many other HCPs in this study reported having witnessed discriminatory behavior in their colleagues, including derogatory language, refusal to use appropriate pronouns, and lack of respect towards LGBTQI people, as reported in previous research (14, 45). These findings demonstrate that LGBTQI patient concerns with disclosure of SOGI status and potential HCP discrimination are a reality, evidenced by accounts of negative judgement and hostility, exclusion of same-gender partners, and cis-heteronormative interventions during cancer care. LGBTQI patients who experienced negative HCP reactions following SOGI disclosure, or experience anti-inclusive care report distress in and disengagement from cancer care (4, 12, 13). This distress may be compounded by previous experiences of discrimination in general health care, which is commonly reported by LGBTQI people, with higher rates of HCP hostility reported toward trans and non-binary people, those from culturally and ethnically diverse backgrounds (9, 70), and people with an intersex variation (85). The ability of HCPs to take up an anti-inclusive subject position and the reluctance of some colleagues to challenge them reflect a broader cultural discourse wherein homophobia and transphobia are still regarded as acceptable (86, 87). In Australia, this is reflected hostile media and public commentary associated with marriage equality (88) and the Safe Schools initiative which aimed to address LGBTQI bullying in primary and secondary schools (89), and government attempts to introduce of a religious discrimination bill, which would legitmate discrimination against LGBTQI people (90). This discourse serves to normalize anti-inclusive and discriminatory practices toward LGBTQI patients and their carers (9), as well as discrimination toward LGBTQI healthcare professionals who disclose their SOGI status at work (91). This may explain why few HCPs in the present study were active in lobbying for LGBTQI inclusivity at a service level, or felt confident in challenging anti-inclusive behavior they witnessed in their colleagues, reinforced by feelings of disempowerment within health system hierarchies.

LGBTQI patients and carers were not passive in response to fears of discriminatory cancer care, demonstrating agency and resistance to invisibility through a process of decision-making and actions. The informal ‘screening’ of HCPs to assess their level of inclusivity and selective disclosure of SOGI status based on HCP response are common strategies adopted by LGBTQI cancer patients (4, 48, 92). The alternative strategy of always disclosing SOGI status to HCPs in the expectation of a positive response demonstrates the intersubjective character of the HCP-patient interaction, with patient self-positioning potentially facilitating HCPs taking up a more inclusive subject position. However, each of these strategies requires additional emotional work by LGBTQI patients and carers, in addition to dealing with the burden of cancer. Disclosure of SOGI status to HCPs who do not adopt an inclusive and affirmative subject position can be a difficult process, involving the emotional work of planning, anticipation of HCP response, and the rehearsal of strategies (9, 48, 71, 92, 93). Patients who are less experienced in SOGI disclosure, such as adolescents and young adults (AYAs), or those who have had previous experiences of HCP or societal discrimination, may be less likely to risk the negative reactions that may follow disclosure (9). This is reflected in lower levels of outness reported by AYAs, TGD and intersex participants in the present study, demonstrating the impact of intersecting identities that produce a matrix of multiple marginalization, in what has been described as a double or triple jeopardy (54). Having to educate HCPs on LGBTQI patient needs and dealing with awkward or ill-informed HCP responses are additional emotional work for LGBTQI patients and carers. HCPs in this study were less confident in their knowledge of the needs of TGD and intersex people (41), and were less likely to adopt reflective and inclusive practice with these groups. This highlights the importance of LGBTQI inclusive and reflective cancer care which creates a place of cultural safety for all patients, whilst recognizing the greater vulnerability and specific concerns of some groups (54).

Non-disclosure of SOGI status can be a place of safety for patients and carers, serving to protect against insensitive or inappropriate HCP responses (9, 48). Indeed, some patients consider their sexual orientation private or irrelevant to cancer care, obviating the need for disclosure (4, 12, 94). However, non-disclosure can be associated with feelings of LGBTQI patient and partner invisibility (4), regret (12) and burden of secrecy (95), as well as absence of specific information relevant to LGBQTI patient needs, which add to the stress of having cancer and to the likelihood of poor psychological wellbeing (96).

## 5 Conclusion

Lack of knowledge or confidence on the part of HCPs in caring for LGBTQI cancer patients has been reported in previous research (1, 2, 36–38, 40). This has led to the development of training programs (25, 26, 97, 98) and publication of practical strategies to facilitate communicative competence in the provision of LGBTQI cancer care (4, 6, 28, 99–103). However, the success of such strategies depends on HCPs being reflective in their practice, acknowledging their own limitations and accepting the necessity of professional training or development, and understanding the complexities and differences within LGBTIQ communities (41). If HCPs position LGBTQI patients as no different from non-LGBTQI patients, or are hostile to LGBTQI people, as was the case with some participants in the present study, such professional development is unlikely to be adopted or effective. These barriers are not immutable, however, as is evidenced by accounts of HCPs in the present study who positioned themselves as knowledgeable and confident in offering inclusive and affirmative care for LGBTQI patients. If oncology HCPs were to adopt this agentive subject position and conceptualize reflective and inclusive care as a routine part of communication with patients, they are more likely to address the needs of their LGBTQI patients (28).

The findings of this study suggest that interventions to improve culturally competent LGBTQI cancer care need to focus on a range of strategies. The materiality of the clinical context needs to be improved in order to facilitate SOGI disclosure and address LGBTQI patient needs. This includes visible indicators of LGBTQI inclusivity in clinics, health service websites, and patient support information; acknowledgement of SOGI status on intake forms, and provision of LGBTQI-specific information on issues such as sexual health, bodily changes, and the concerns of transgender and intersex people (22, 75, 104). Onward referral services are needed to provide support for HCPs when patients require LGBTQI-specific expert interventions (28). Clinical management teams and clinical mentors also need to acknowledge the importance of addressing the needs of LGBTQI patients, and support the development of HCP communicative competence (105), facilitating HCPs to adopt an inclusive and reflective subject position. Specific training in offering inclusive and affirmative cancer care as part of basic communication training and ongoing professional development is essential (25, 28). Such programs can increase HCP confidence, challenge homophobic and transphobic stereotypes and increase the likelihood of LGBTQI patients receiving inclusive and affirmative cancer care.

Derogatory constructions of LGBTQI patients, or representations of LGBTQI patients as no different from any other patient, need to be challenged in order to undermine discursive strategies that exonerate HCPs from offering inclusive and affirmative care. There is a need for HCPs to be aware of the potential vulnerability of LGBTQI patients, in particular difficulties in SOGI disclosure and the impact of invisibility in health care. There is also a need for awareness that HCPs have responsibility for facilitating SOGI disclosure with their patients, as many LGBTQI patients are too fearful to disclose, or are concerned that they will receive negative responses. Providing equitable care to LGBTQI cancer patients and their carers is a human rights issue. We know what patients want, and we know the barriers to provision of inclusive and affirmative person-centered LGBTQI cancer care. It is time to translate this knowledge into education and training for all oncology HCPs and to ensure there are appropriate and targeted resources and information for LGBTQI patients and their carers.

## The Out With Cancer Study Team Members

Chloe Parton^1^, Antoinette Anazodo^2^, Fiona E. J. McDonald^3^, Katherine Boydell^4^, Kerry H. Robinson^5,^. Gary W. Dowsett^6^, Suzanne Chambers^7^, Martha Hickey^8^, Ian D. Davis^9^, Cristyn Davies^10^ and Felix Delhomme^11^

^1^ School of Health, Te Herenga Waka – Victoria University of Wellington, Wellington, New Zealand

^2^Kids Cancer Centre, Sydney Children’s Hospital and School of Women’s and Children’s, University of New South Wales, Sydney, Australia

^3^Canteen and Faculty of Medicine and Health, The University of Sydney, Sydney, Australia

^4^Black Dog Institute, University of New South Wales, Sydney, Australia

^5^School of Social Sciences and Translational Health Research Institute, Western Sydney University, Sydney Australia

**^6^** Australian Research Centre in Sex, Health and Society, La Trobe University, Melbourne, Australia

^7^Faculty of Health Sciences Australian Catholic University, Brisbane, Australia

^8^Department of Obstetrics and Gynaecology, University of Melbourne and the Royal Women’s Hospital, Melbourne, Australia

^9^Eastern Health Clinical School, Monash University and Eastern Health, Melbourne, Australia.

^10^Specialty of Child and Adolescent Health, Faculty of Medicine and Health, University of Sydney, Australia; and School of Social Sciences and Psychology, Western Sydney University

^11^ACON, Sydney, Australia

## Data Availability Statement

The raw data supporting the conclusions of this article will be made available by the authors, without undue reservation.

## Ethics Statement

The studies involving human participants were reviewed and approved by Western Sydney University Human Research Ethics Committee (H12664). Written informed consent to participate in this study was provided by the participants’ legal guardian/next of kin.

## Author Contributions

JU and JP designed the study and prepared the application for funding, in collaboration with The Out with Cancer Study Team members. Data were collected and coded by RP, KA, and AH. JU and RP conducted analysis of the data in collaboration with AH, JP, and KA. The Out with Cancer Study Team made critical commentary on the coding, the analytic plan and on the written paper. All authors contributed to the article and approved the submitted version.

## Funding

This study was funded by the Australian Research Council Linkage Program grant [LP170100644], the Cancer Council New South Wales, and Prostate Cancer Foundation Australia, with in-kind support provided by National LGBTI Health Alliance, ACON, Breast Cancer Network Australia, Sydney Children’s Hospital Network, and Canteen.

## Conflict of Interest

The authors declare that the research was conducted in the absence of any commercial or financial relationships that could be construed as a potential conflict of interest.

## Publisher’s Note

All claims expressed in this article are solely those of the authors and do not necessarily represent those of their affiliated organizations, or those of the publisher, the editors and the reviewers. Any product that may be evaluated in this article, or claim that may be made by its manufacturer, is not guaranteed or endorsed by the publisher.
